# Transcriptome analysis of *Pseudostellaria heterophylla* in response to the infection of pathogenic *Fusarium oxysporum*

**DOI:** 10.1186/s12870-017-1106-3

**Published:** 2017-09-18

**Authors:** Xianjin Qin, Hongmiao Wu, Jun Chen, Linkun Wu, Sheng Lin, Muhammad Umar Khan, Mohammad Reza Boorboori, Wenxiong Lin

**Affiliations:** 1 Key Laboratory for Genetics, Breeding and Multiple Utilization of Crops, Ministry of Education/College of Crop Science, Fujian Agriculture and Forestry University, Fuzhou, 350002 People’s Republic of China; 2 Key Laboratory of Crop Ecology and Molecular Physiology (Fujian Agriculture and Forestry University), Fujian Province University, Fuzhou, 350002 People’s Republic of China; 3 Fujian Provincial Key Laboratory of Agroecological Processing and Safety Monitoring, College of Life Sciences, Fujian Agriculture and Forestry University, Fuzhou, 350002 Fujian People’s Republic of China

**Keywords:** *Pseudostellaria Heterophylla*, *Fusarium oxysporum*, Transcriptome, Consecutive monoculture problems, Calcium signal

## Abstract

**Background:**

*Pseudostellaria heterophylla* (*P. heterophylla*), a herbaceous perennial, belongs to *Caryophyllaceae* family and is one of the Chinese herbal medicine with high pharmacodynamic value. It can be used to treat the spleen deficiency, anorexia, weakness after illness and spontaneous perspiration symptoms. Our previous study found that consecutive monoculture of *Pseudostellaria heterophylla* could lead to the deterioration of the rhizosphere microenvironment. The specialized forms of pathogenic fungus *Fusarium oxysporum f.Sp. heterophylla* (*F. oxysporum*) in rhizosphere soils of *P. heterophylla* plays an important role in the consecutive monoculture of *P. heterophylla*.

**Results:**

In this study, *F. oxysporum* was used to infect the tissue culture plantlets of *P. heterophylla* to study the responding process at three different infection stages by using RNA-sequencing. We obtained 127,725 transcripts and 47,655 distinct unigenes by de novo assembly and obtained annotated information in details for 25,882 unigenes. The Kyoto Encyclopedia of Genes and Genomes pathway analysis and the real-time quantitative PCR results suggest that the calcium signal system and WRKY transcription factor in the plant-pathogen interaction pathway may play an important role in the response process, and all of the WRKY transcription factor genes were divided into three different types. Moreover, we also found that the stimulation of *F. oxysporum* may result in the accumulation of some phenolics in the plantlets and the programmed cell death of the plantlets.

**Conclusions:**

This study has partly revealed the possible molecular mechanism of the population explosion of *F. oxysporum* in rhizosphere soils and signal response process, which can be helpful in unraveling the role of *F. oxysporum* in consecutive monoculture problems of *P. heterophylla*.

**Electronic supplementary material:**

The online version of this article (10.1186/s12870-017-1106-3) contains supplementary material, which is available to authorized users.

## Background

The consecutive monoculture problem is a popular phenomenon to many crops in modern agriculture production [1–4]. It is extremely serious in the production of Chinese herbal medicine. Some researchers point out that about 70% of medicinal plant species with tuber roots have various degrees of consecutive monoculture problems, including *P. heterophylla*, *Rehmannia glutinosa*, *Panax notoginseng* and *Angelica sinensis*, etc. [5]. This issue not only influence the medicinal efficacy, but also cause enormous economic losses. Our oriented field experiment has been carried out on the site located in one of the geoauthentic growing area (Zherong city, Fujian province, China) of *P. heterophylla* for more than three years, we found that the consecutive monoculture of *P. heterophylla* causes yield-decline of the tuberous roots and the aggravation of soil-borne disease [6].

Nevertheless, most of previous studies on this problem mainly focused on the underground part (the rhizosphere of the plant). These results indicated that the successive monoculture leads to decreased bacteria population in rhizosphere soil, but the reverse is true in the case of fungus population [7, 8]. The differential changes in the composition of microbial flora have been considered to be mediated by the accumulated root exudates from medicinal plants being continuously monocultured in the same field [9, 10]. It has been reported that a wide variety of compounds exudated from the roots of the monocultured plants, could be degraded or transformed to other allelochemicals, thereby orchestrate the soil microbial community in their immediate vicinity. Due to this reason, chemical and physical properties of the soil alters, consequently retard the growth and development, which ultimately leads to the yield and quality decline of the medicinal plants [6, 9–13]. Badri et al. [14] found that different groups of compounds might impact the soil microbe composition at various levels and the phenolic compounds act as specific substrates or signaling molecules for a specific microbe. More and more evidences indicate that *F. oxysporum* plays a notorious role in the consecutive monoculture of *P. heterophylla*. In our research findings, the denaturing gradient gel electrophoresis (DGGE) analysis of fungi in the different soil of the consecutive monoculture of *P. heterophylla* showed that the pathogenic fungus, *F. oxysporum* was the main causal organism of soil-born diseases, such as the root rot disease and Fusarium wilt of *P. heterophylla* plants in the continuous cropping system. The further studies suggested that some phenolic acids exudated from the roots of monocultured *P. heterophylla* plants were able to promote the growth of *F. oxysporum f.Sp. heterophylla* mycelium and spore production [9, 10]. Wu et al. [13] also discovered that mixed phenolic acid could promote mycelial growth, sporulation and toxin production of pathogenic *F. oxysporum*, implying that the allelochemicals including phenolic acids, organic acids, etc., released in the root exudates by the monocultured plants might mediate the differential changes of microbial flora in rhizosphere soil under monoculture cropping system.

As mentioned before, the study on rhizosphere biology of monocultured *P. heterophylla* has been an important topic recently, but the researches on the relationship between *P. heterophylla* and the main pathogenic fungus were rarely reported on the molecular level. With the rapid development of the sequencing technologies, these technologies have been widely used in the fields of biology to provide fundamental insight into biological processes. The transcriptome was used to investigate the petal senescence in four developmental stages in *Gardenia jasminoides* [15], other researchers also chose different organs and tissues to study the specifically expressed genes or promoters, and to complete the functional annotation of differentially expressed genes [16]. Moreover, next generation sequencing tools were also used to analyze non model ectomycorrhizal plant-fungal interactions that can contribute to find the “symbiosis tool kits” and can better define the role of each partner in the mutualistic interaction [17]. Besides, De novo characterization of *Rehmannia glutinosa Libosch* leaf transcriptome was analyzed to get the most important resource for further investigating the cause of replanting disease and developing methods to control or reduce its harmful effects [18]. Since the RNA sequencing (RNA-Seq) allows unbiased quantification of expression levels of transcripts with a higher sensitivity, which can be an effective way to explore the differentially expressed genes and signal transduction process in *P. heterophylla* responding to the infection of *F. oxysporum*.

When we want to know the correlations between microorganisms and plants, the signaling in the rhizosphere becomes particularly important. The latest research has divided the signaling in the rhizosphere into three categories: (a) intra and interspecies signaling among microorganisms, (b) plant-microorganism signaling, and (c) microorganism-plant signaling [19]. Since little is known about the microorganism-plant signaling between pathogenic *F. oxysporum* and *P. heterophylla*. Guo et al. [20] analyzed the calcium signaling system of *Rehmannia glutinosa* in the field and pointed out that the calcium-dependent signaling pathways took part in the consecutive monoculture problem of *Rehmannia glutinosa*. Importantly, this pathway also plays well-established roles in plant innate immunity and calcium serves as a versatile messenger in many adaptation and developmental processes in plants [21]. Furthermore, the Ca^2+^ sensors are encoded by complex gene families and form intricate signaling networks in plants to enable specific and flexible information processing. Meanwhile, these signals need to be detected, decoded and transmitted by a complex calcium-dependent signaling network, including protein-protein interactions and phosphorylation cascades to trigger subsequent downstream transcriptional reprogramming [22, 23]. The plant decoding Ca^2+^ instrument mainly includes different families of Ca^2+^ sensors like Calmodulins (CaM), Calmodulin-like proteins (CMLs), Ca^2+^-dependent protein kinases (CDPKs), Calcineurin B-like proteins (CBLs) and their interacting kinases (CIPKs) [21]. Meanwhile, the cyclic nucleotide gated channels (CNGCs) also have been found contributing to Ca^2+^ conductance in pathogen defense signal transduction [24]. Additionally, Zhu et al. [25] found numbers of genes encoding transcription factors (TFs) in the *F. oxysporum*-infected *Arabidopsis* and pointed out that the majority of these TFs belong to the WRKY, ERF, MYB families, etc. Defense-associated genes are normally regulated positively or negatively by TFs, TFs are direct or indirect targets of various signal transduction pathways [26, 27]. Therefore, the main purpose of this study is to analyze the expression pattern of the succedent downstream defense-associated genes and transcription factors by using RNA-Seq technology. Exploring the signal transduction process is helpful to insight into the underlying molecular mechanism of the replanting disease in the continuous cropping system of the Chinese medicinal plants.

## Results

### Abundance of *F. oxysporum* in different rhizosphere soil and its infection to the plants

**Fig. 1:**
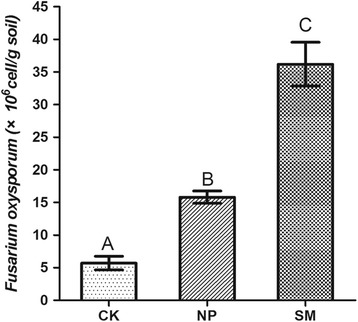
Quantification of *F. oxysporum* in three different soil samples. CK: Control soil; NP: newly planted soil; SM: two-year monoculture soil. Different superscripts in the column indicate significant differences with each other (*P* ≤ 0.01,*n* = 4)

**Fig. 2:**
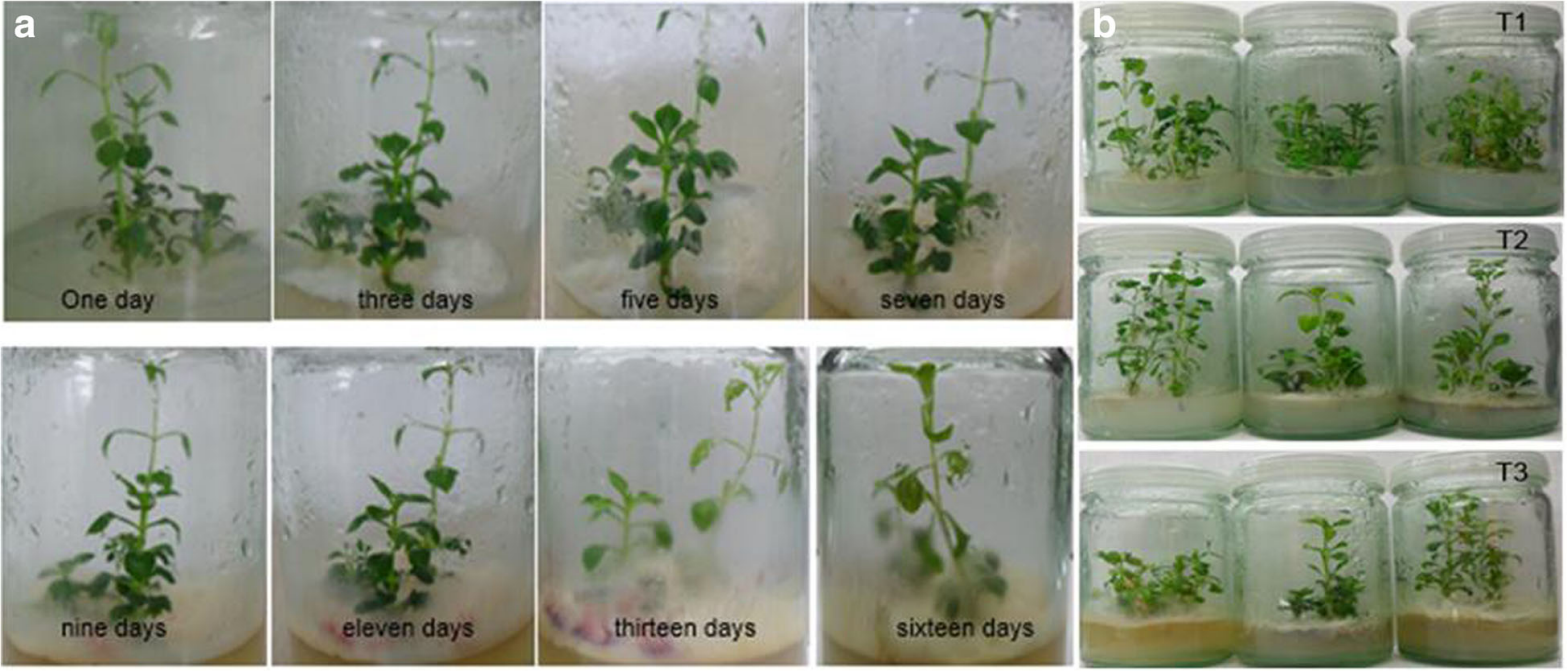
The *P. heterophylla* seedlings infected by *F.oxysporum* in different days. (**a**) The *P. heterophylla* seedlings infected by *F. oxysporum* in different days, (**b**) the first stage, the plants without adding the *F. oxysporum* (T1), the second stage, the plants infected for 8 days (T2), the third stage, the plants infected for 13 days (T3)

We used the quantitative real time PCR (qRT-PCR) in the analysis of the abundance of *F. oxysporum* in different rhizosphere soil, the results indicated that the abundance of *F. oxysporum* in two-year monoculture soil (SM) was much higher than the newly planted soil (NP) and control soil (CK), meanwhile the amount of *F. oxysporum* in two-year monoculture soil (SM) was 2.3 times higher than that in newly planted soil (NP), and all of the differences reached an extremely significant level (*P* ≤ 0.01) (Fig. 1). These results implied that *F. oxysporum* may play an important role in the consecutive monoculture problems of *P. heterophylla*. Since we inoculated the pathogen on the medium about 2–3 cm away from the roots of plants, the pathogenic fungi *F. oxysporum* initiated to contact with roots about three days later, the leaves start to turn yellow about one week later and the basal part of the stem was covered with *F. oxysporum* and the plants died two weeks later (Fig. 2a). Three different stages were selected for RNA-Sequencing: the first stage the plants without adding the *F. oxysporum* (T1), the second stage with the plants infected for 8 days (T2) and the third stage with the plants infected for 13 days (T3) (Fig. 2b).

### Illumina sequencing and functional genes annotated analysis of *Pseudostellaria heterophylla*

In order to obtain more detailed information about *P. heterophylla* transcriptome, a cDNA library was constructed from RNA isolated from the whole plants of the three samples and sequenced on the Illumina Hiseq2000™. According to the sequencing results, we filtered the raw data to insure that those reads with less than 20 bases were below than 20% and the nitrogen content was less than 5%, besides, we also removed the ribosome RNA and the contaminated data from the raw data to obtain the highest quality data. After this, all the Q30 of the bases were significantly higher than 93.34%. Since there was no assembled or annotated *P. heterophylla* genomic sequences that could be used for transcript assembly; trinity de novo assembler was used to assemble all the trimmed reads with optimized K-mer length of 25. We obtained 2,583,941 contigs from the overlap information of different reads and then clustered analysis these contigs based on the similarity between the pair-end information and the contigs. After that, we acquired the transcripts through the partial assembling and screened out the main transcripts as the unigene. Finally, the total reads from the three samples were assembled into 127,725 transcripts and 47,655 unigenes with the N50 length of them were 2274 and 1490 respectively. The statistical results of the assembly contigs, transcripts and unigenes in different length range are shown in Additional file 1: Table S1, the mean lengths of the contigs, transcripts and unigenes were 57.84, 1490.74 and 828.90, respectively.

Bioinformatics tools such as BLAST were used to compare the unigene sequences to the NR (NCBI non-redundant protein sequences), COG (Clusters of Orthologous Groups of proteins), Swiss-Prot (A manually annotated and reviewed protein sequence database), KO (KEGG Ortholog database), GO (Gene Ontology) databases. Finally, 25,882 unigenes got their annotated information in details as shown in the Additional file 2: Table S2.

### Gene ontology (GO) annotation and clusters of Orthologus groups (COG) annotation of *P. heterophylla* transcripts

**Fig. 3:**
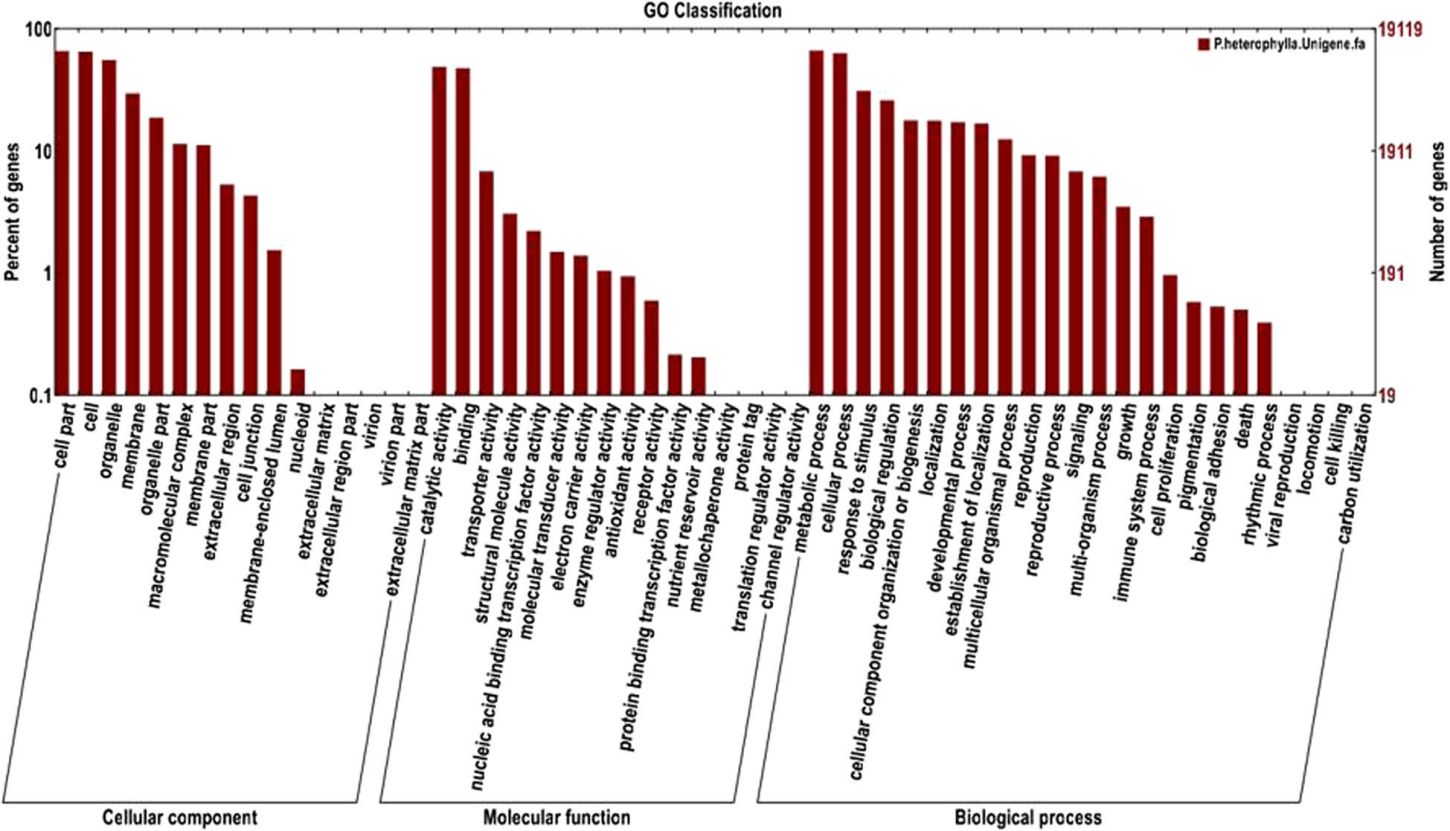
GO classification of the total assembled unigenes. The left side and the right side of the panel show the percentage of genes and the number of genes that are classified in the corresponding term, respectively

The unigenes of *P. heterophylla* were classified into three categories according to the Gene Ontology annotation results, namely “Cellular Component” and “Molecular Function” and “Biological Process” (Fig. 3). In the category of “Cellular Component”, most of the unigenes belonged to the “cell part”, “cell”, “organelle”, “membrane”, “organelle part”, “macromolecular complex”. While in the category of the “Molecular Function”, these unigenes with the function of “catalytic activity”, “binding”, “transporter activity” occupied the main part. Furthermore, some of the “Biological Process”, such as “metabolic process”, “cellular process”, “response to stimulus”, “biological regulation” etc. were the ascendant terms. Moreover, “developmental process”, “establishment of localization”, “multicellular organismal process”, “reproduction”, “reproductive process” and “signaling” also took a large proportion of the “Biological Process”, but when it comes to the differentially expressed unigenes (DEGs) at different stages (Additional file 3: Figure S1), there still existed small differences. The intracellular part, especially the intracellular organelle and the membrane take up significant advantages in a cellular component except the cell and cell part. Meanwhile, the DEGs with the function of transferase, hydrolase, ion or protein binding, oxidoreductase and substrate-specific transporter may have a more crucial molecular function in the infection process.

**Fig. 4:**
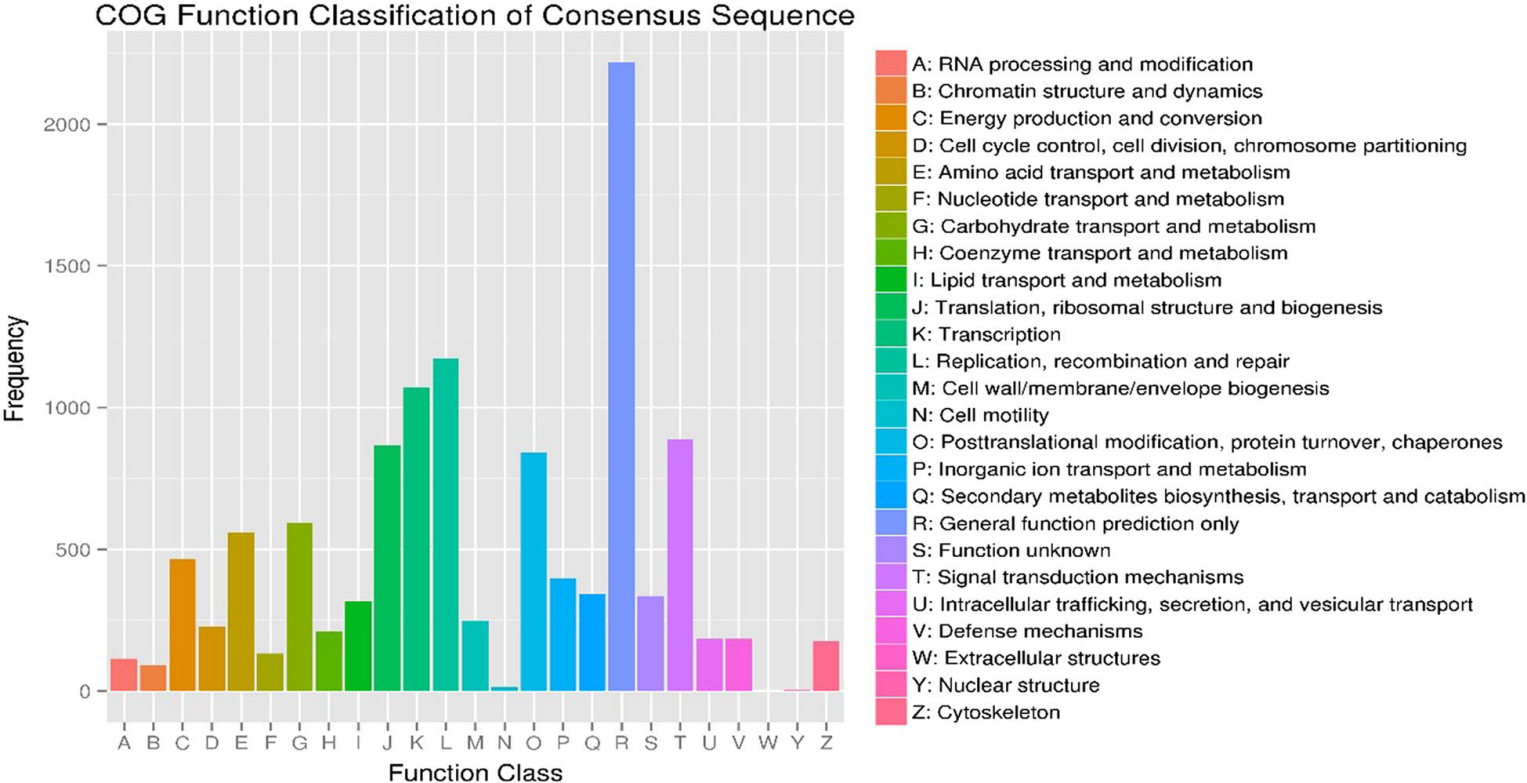
COG annotation of the total assembled unigenes. The left side and the right side of the graph show the frequency number and the classified COG categories, respectively

We searched all of the 11,648 unigenes against the COG databases and found that all the consensus sequences were classified into 24 categories. As shown in the COG annotation (Fig. 4), the “General function prediction only” has the largest group with 2216 unigenes followed by “Replication, recombination and repair” (1174 unigenes), “Transcription” (1072 unigenes), “Signal transduction mechanisms” (887 unigenes), “Translation, ribosomal structure and biogenesis” (866 unigenes), “Posttranslational modification, protein turnover, chaperones” (842 unigenes), “Carbohydrate transport and metabolism” (593 unigenes), “Amino acid transport and metabolism” (561 unigenes), “Energy production and conversion” (466 unigenes). When it comes to the differential expression unigenes, the “General function prediction only” still own the most differentially expressed unigenes at different stages. However, “Signal transduction mechanisms”, “Transcription” and “Posttranslational modification, protein turnover, chaperones” were the largest groups to have the most number of differential expression unigenes during the early stage of the infection (Additional file 4: Figure S2) followed by “Carbohydrate transport and metabolism”, “Replication, recombination and repair” and “Amino acid transport and metabolism”; In the later stage of the infection, the “Posttranslational modification, protein turnover, chaperones”, “Replication, recombination and repair” and “Signal transduction mechanisms” obviously play a more important role in this period than the “Transcription”, “Carbohydrate transport and metabolism” and “Amino acid transport and metabolism” (Additional file 5: Figure S3). While in the group between T2 and T3 (Additional file 6: Figure S4), the “Amino acid transport and metabolism”, “Carbohydrate transport and metabolism” and “Replication, recombination and repair” become even more important than the following “Posttranslational modification, protein turnover, chaperones”, “Signal transduction mechanisms”.

### DEGs response to infection of *F. oxysporum* and the Kyoto encyclopedia of genes and genomes (KEGG) pathway analysis

The unigenes that were differentially expressed in pairs of two consecutive stages response to the infection of the *Fusarium oxysporum* are shown in Table 1. When we compared the relative expression level of unigenes between the T1 and T2 stage, T1 stage was chosen as the control group, a total of 1905 unigenes were differentially expressed, 1242 unigenes of which were up-regulated and 663 were down-regulated. Among them, there were 1187 unigenes which were differentially expressed only in these two developmental stages but not in the other consecutive stages (Additional file 7: Figure S5). When the plants were infected for thirteen days (T3), we found that 1455 unigenes were differentially expressed from the T1 stage, of which 1274 unigenes were up-regulated and the rest of them were down-regulated, and what’s more, about 255 unigenes just differentially expressed between T1 and T3. Moreover, in the other group, we found that 1047 DEGs between T2 and T3, which were also divided into two parts, the up-regulated and down-regulated unigenes were 698 and 376, respectively. Furthermore, we were also able to obtain some common DEGs in different groups, 484 DEGs could be found only in group T1 and T2 and group T1 and T3, meanwhile, 507 DEGs were identified just both in group T1 and T2 and in group T2 and T3, and only 25 DEGs were in common in group T1 and T2 and group T2 and T3. Another important thing is that 209 common DEGs came out in three different stages.

**Table 1:** The differential expression unigenes statistical analysis of different stages

Type	Number	up	down
T1 vs T2	1905	1242	663
T1 vs T3	1455	1274	181
T2 vs T3	1047	698	376

**Fig. 5:**
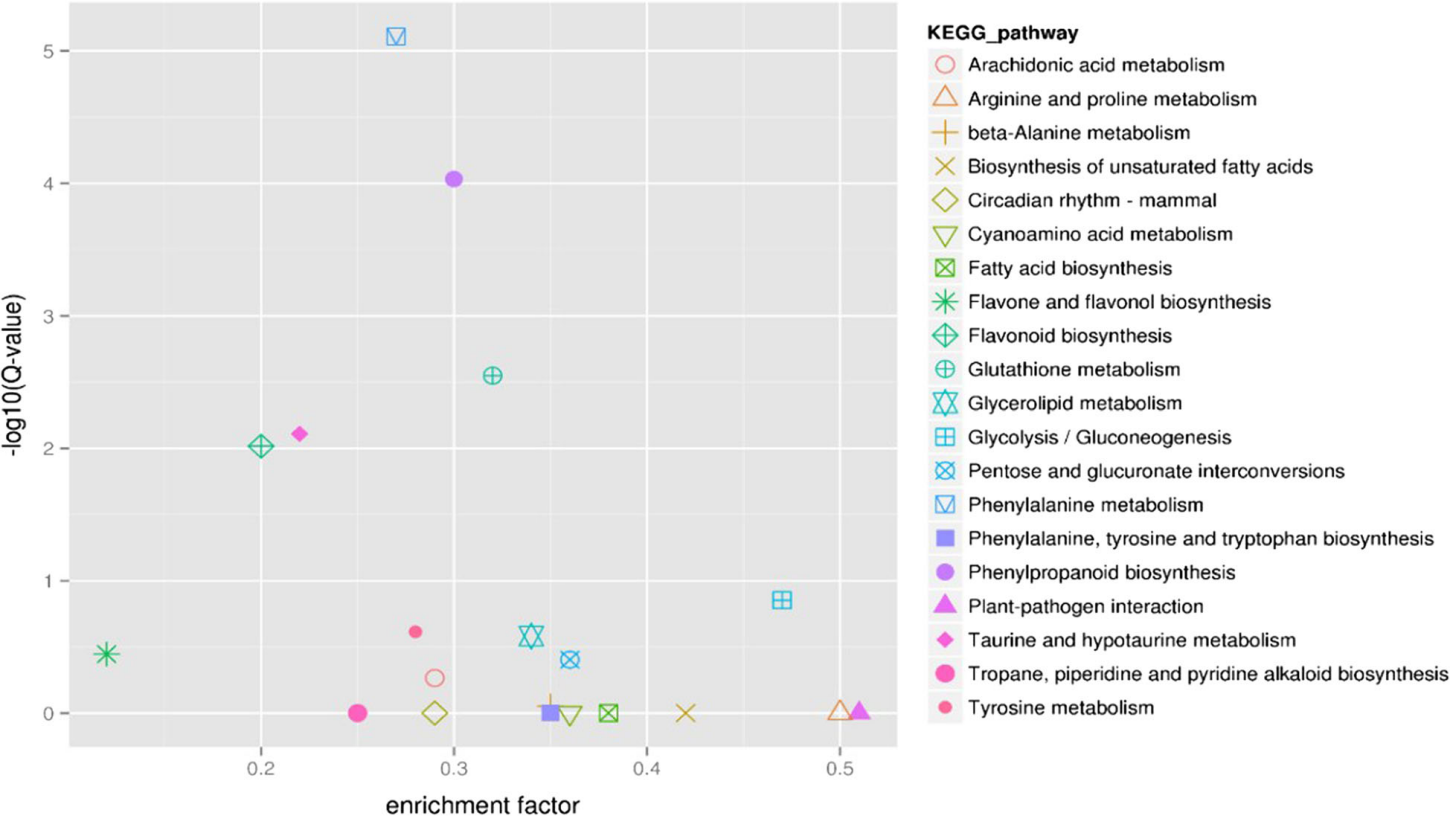
The KEGG pathway enrichment scatter diagram of the differential expression genes between T1 stage and T2 stage. The X-axis shows the different enrichment factor of different pathways, and the enrichment factor stand for the ratio between the proportion of DEGs in one pathway and the proportion of all the annotated unigene in the same pathway, the Y-axis shows the different value of “–log_10_^(Q-value)^”, the Q-value stands for *P*-value after multiple hypothesis testing. The most significant enrichment of twenty pathways was shown in different color and shape in the left of the picture

From the Kyoto Encyclopedia of Genes and Genomes scatter diagram between T1 and T2 (Fig. 5), we chose twenty of the most significant enrichment pathways to show the differential expression unigenes in different pathways, of which “Phenylalanine metabolism”, “Phenylpropanoid biosynthesis”, “Glutathione metabolism”, “Taurine and hypotaurine metabolism” and “Flavonoid biosynthesis” show the extremely significant difference (Q-value < 0.01). Besides, these pathways such as “Plant-pathogen interaction”, “Arginine and proline metabolism” and “Glycolysis/Gluconeogenesis” with the highest enrichment factor may also play an important role in response to the infection of the *F. oxysporum*.

We used the qRT-PCR to validate the expression of the DEGs during different stages, the DEGs were randomly selected from T1 and T2 but also T1 and T3, which included both the up-regulated and the down-regulated expression genes. To describe the qRT-PCR results more directly and clearly in accordance the RNA sequencing results, we used the “2^(−∆∆C^_T_^)^” value to represent the expression of the unigenes to correspond with the RPKM value which can represent the RNA sequencing results of the differentially expressed unigenes. The expression of the qRT-PCR results of the differential expression unigenes were assumed as one at T1 stage, those qRT-PCR results with the value between zero and one means that the unigene was down-regulated in that stage. While the other results, which had a value bigger than one, implying that the unigene was up-regulated. By our research findings, the qRT-PCR experiment results met well with the RNA Sequencing results, indicating that the differentially expressed unigenes screened from the RNA-Sequencing are reliable and available (Additional file 8: Figure S6).

### Roles of the DEGs related to calcium signal in the plant-pathogen interaction pathway

**Fig. 6:**
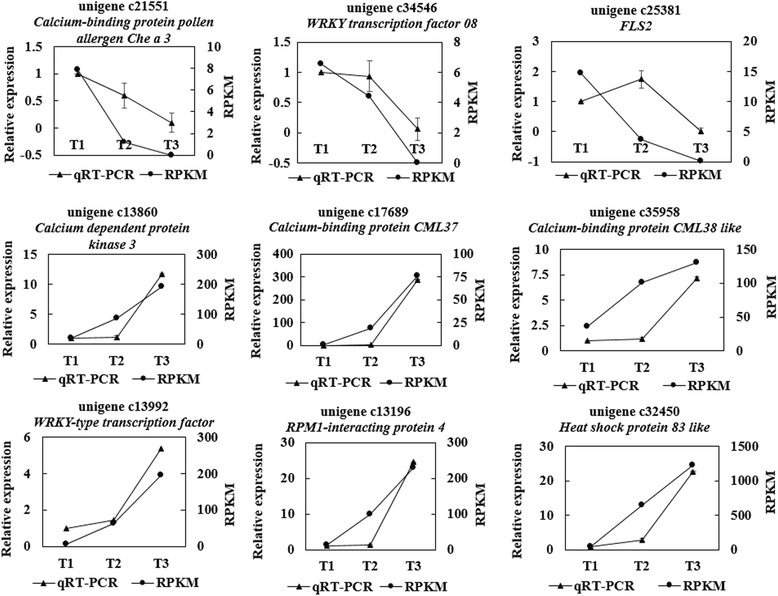
Analysis of the differential expression unigenes selected of the Plant-Pathogen interaction pathway with RNA-Seq and qRT-PCR. The lines with triangle indicate the qRT-PCR results with the “2^(−∆∆C^_T_^)^” value corresponding to the primary axis on the left side and the lines with dot show the RNA-Seq results with the RPKM value corresponding to the secondary axis on the right side. The expression quantity of all the unigenes was set as one at T1 stage, and T1, T2, T3 stands for different stage respectively

According to the KEGG pathway analysis result, the plant-pathogen interaction pathway had the highest enrichment factor (Fig. 5), we found most of the differential expressed unigenes in this pathway were up-regulated except for the LRR receptor-like serine/threonine-protein kinase *FLS2* and *WRKY transcription factor 08*. The pathogen-associated molecular patterns mediates the unigenes which are closely contacted with the calcium signal, such as *CDPK*, *CML*, *CaM*. These genes and the WRKY transcription factor were up-regulated both at T2 and T3 stage when they were compared with that at T1 stage (Additional file 9: Figure S7). We used qRT-PCR to analyze these DEGs related to the plant-pathogen interaction pathway, the results suggested that *Calcium dependent protein kinase 3* (unigene c13860, *CDPK3*), *Calcium-binding protein CML37/38* (unigene c17689, *CML37*; unigene c35958, *CML38*), *WRKY-type transcription factor* (unigene c13992, *WRKY*), *RPM1-interacting protein 4* (unigene c13196, *RIN4*) and *heat shock protein 83-like* (unigene c32450, *Hsp83*) were up-regulated both at T2 stage and T3 stage. But not all of them were up-regulated, some of the unigenes such as the *Calcium-binding pollen allergen Che a 3* (unigene c21551) and *WRKY transcription factor 08* (unigene c34546, *WRKY08*) were all down-regulated at T2 stage and T3 stage. Besides, the *LRR receptor-like serine/threonine-protein kinase FLS2* (unigene c25381, *FLS2*) was up-regulated at T2, but down-regulated at T3 stage, showing slightly different from the RNA-Seq result at T2 stage (Fig. 6), the primers of these unigenes were shown in the Additional file 10: Table S3.

**Fig. 7:**
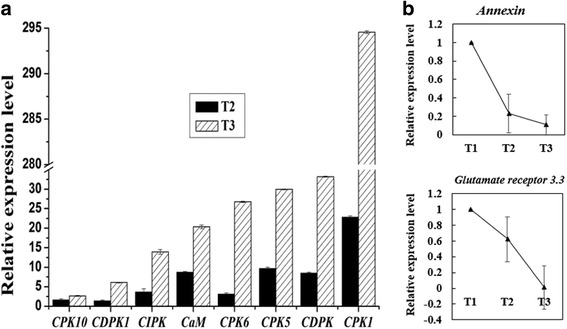
Relative expression level of the calcium signal related genes in different infection stages. **a** The genes includes *CPK10* (*unigene c19133*), *CDPK1* (*unigene c28761*), *CIPK*, *CaM*, *CPK6* (*unigene c32189*), *CPK5* (*unigene c34375*), *CDPK* (*unigene c26726*), *CPK1* (*unigene c14000*); **b** The line chart contains *Annexin* (*unigene c26955*) and *Glutamate receptor 3.3* (*unigene c32527*), the expression quantity of the last two unigenes were set as one at T1 stage. T1, T2 and T3 stand for different stage respectively

Moreover, other unigenes related to the calcium signal system were analyzed on the basis of the RNA-Seq results. These unigenes, principally include the CBL-interacting protein kinase, Calcium binding protein, Calcium transporting ATPase, Annexin, Glutamate receptor etc. (Additional file 11: Figure S8). The unigenes of the calcium signal decoding system, including *Calcium dependent protein kinase gene* (*unigene c26726*, *unigen c31289*, *unigene c19133*, *unigene c13860*), *CBL-interacting protein kinase gene* (*unigene c33227*, *unigene c31920*, *unigene c33951*, *unigene c13791*) and *Calcium binding protein gene* (*unigene c17699*, *unigne c36329*, *unigne c17689*, *unigne c35958*) were up-regulated both at T2 and T3 stage. In contrast, some of the unigenes of the calcium signal encoding system were down-regulated, such as *Glutamate receptor gene* (*unigene c35532*, *unigene c32236*, *unigene c28906*, *unigne c35578*), *Annexin gene* (*unigene c36074*, *unigene c26955*), *Calcium transporting ATPase gene (unigene c32998 and unigene c35597)*, but it is not suitable for all of the unigenes. Other unigenes, for example, *Annexin gene* (*unigene c13258*, *unigene c27953*) and *Calcium transporting ATPase gene* (*unigene c29327*, *unigene c33245*) were up-regulated from T1 stage to T2 stage but then down-regulated from T2 stage to T3 stage. The qRT-PCR results (Fig. 7a) showed that the *CPK10* (*unigene c19133*), *CDPK1* (*unigene c28761*), *CPK6* (*unigene c32189*), *CPK5* (*unigene c34375*), *CDPK* (*unigene c26726*), *CPK1* (*unigene c14000*), *CIPK* and *CaM* were up-regulated both at T2 stage and T3 stage (T1 stage is chosen as the control), these genes were up-regulated from T2 stage to T3 stage too. The *Annexin* (*unigene c26955*) and *Glutamate receptor 3.3* (*unigene c32527*) were down-regulated from T1 stage to T3 stage orderly (Fig. 7b). The primers are shown in the Additional file 10: Table S3.

**Fig. 8:**
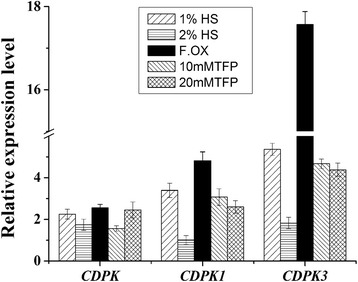
The effects of different concentrations of calcium channel blocker to the infected *P. heterophylla*. The related unigenes were *CDPK* (*unigenec26726*), *CDPK1* (*unigene c14000*) and *CDPK*3 (*unigene c13860*). The plants without any infection by the pathogen and treated with double distilled water were chosen as the control, the F.OX refers to the plants infected by *F. oxysporum* and sprayed with ddH_2_O in the same way as the control, the other four treatments of 1% HS, 2% HS, 10 mM TFP and 20 mM TFP refers to the plants which were treated with different concentrations of calcium channel blocker after they were infected by *F. oxysporum*

Different concentrations of calcium channel blocker heparin sodium (HS) and trifluoperazine (TFP) were used to confirm the function of the unigenes related calcium signal in this process, these blockers were sprayed on the plants which were suffering from the infection of *Fusarium oxysporum*. The plants without any infection by *Fusarium oxysporum* and treated with the sterile double distilled water were chosen as the control. The plants infected by *Fusarium oxysporum* and treated in the same way as the control were named as the F. OX. The expressions of genes of all the treatments were compared with the control. As illustrated by figure (Fig. 8), the qRT-PCR results showed that both *CDPK* (unigene c26726), *CDPK1* (unigene c14000) and *CDPK*3 (unigene c13860) were distinctly up-regulated under the infection by the *F. oxysporum f.Sp. heterophylla*, these unigenes expressed 2.6, 4.8 and 17.6 times higher than that in the control respectively. However, after treated with the 1% HS and 2% HS, *CDPK (unigene c26726)* was up-regulated only 2.3 and 1.7 times higher than the control, *CDPK1 (unigene c14000)* expressed only 3.4 and 1.0 times higher than the control, and *CDPK3 (unigene c13860)* expressed only 5.4 and 1.8 times higher than the control. Additionally, the samillar trend was observed in the treatment with 10 mM TFP and 20 mM TFP. The qRT-PCR results further confirmed that the calcium channel blockers were able to weaken the expression of some *CDPK gene* and the inhibition rate became more distinct when the dosage of the blocker were higher.

### The changes of calcium concentration of the *P. heterophylla* root tip after the infection of *F.oxysporum*

**Fig. 9:**
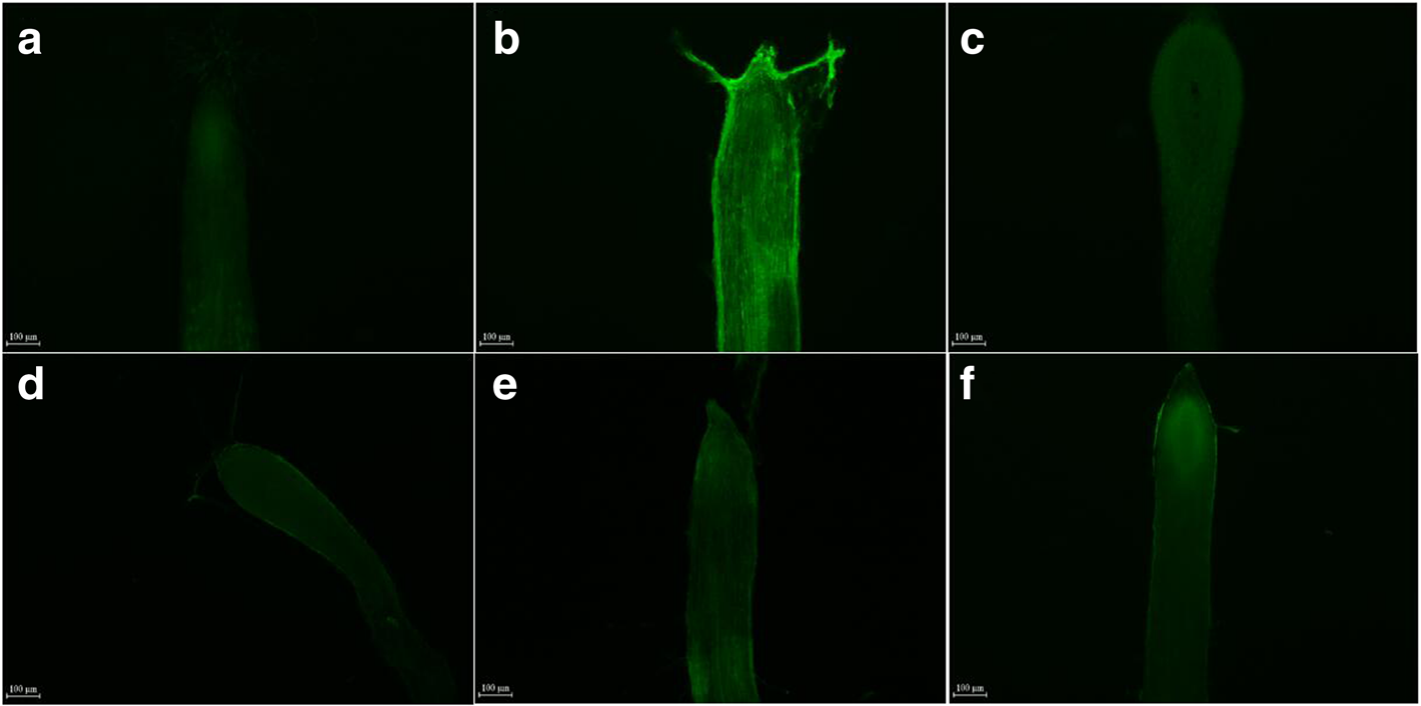
The changes of calcium concentration of the *P. heterophylla* root tip after the infection by *F.oxysporum*. **a** The control without adding the fungal suspension, but sprayed with sterile water (CK), **b** the treatment infected by *F.oxysporum* but without spraying the blocker (F.OX), **c** the root tip infected by *F.oxysporum* and sprayed with 20 mM Trifluoroperazine Dihydrochloride (F.OX + 20 mM TFP), **d** the root tip infected by *F.oxysporum* and sprayed with 20 mM Verapamil (F.OX + 20 mM Verapamil), **e** the root tip infected by *F.oxysporum* and sprayed with 20 mM Lanthanum (III) chloride-anhydrous (F.OX + 20 mM LaCl_3_), **f** the root tip infected by *F.oxysporum* and sprayed with 2% HS (F.OX + 2% HS). All of the figures were taken under ten times fluorescence microscope with the white 100 μm ruler line. The deeper the intensity of the fluorescence suggests the higher concentration of the calcium ion in the root tips

We also used the Fluo-3 AM probe to detect the changes of calcium concentration of the *P. heterophylla* root tip after the infection by *F.oxysporum*, we were abled to show that after the infection of the pathogen the fluorescence intensity was significantly enhanced (Fig. 9a, b). This result suggests that the concentration of calcium ion in the root tip significantly increases after the infection as compared to the root tip without adding the pathogen. Moreover, the use of four different kinds of calcium channel blockers (20 mM TFP, 20 mM Verapamil, 20 mM LaCl_3_ and 2% HS) can reduce the fluorescence intensity (Fig. 9c-f). All of these results suggest that calcium signal events do take part in this process.

### The relative quantitative analysis of the key genes in phenylpropaniod biosynthesis pathway and the quantitative analysis of the phenolics from the tissue culture medium of *P. heterophylla*

**Fig. 10:**
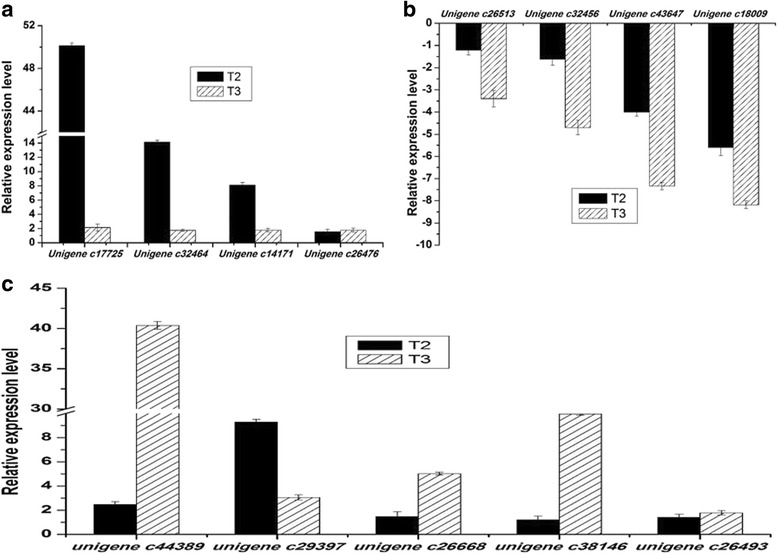
The qRT-PCR analysis of the key genes in the phenylpropaniod biosynthesis pathway and genes related to cell death. **a** *Unigene c17725* and *unigene c32464* belongs to *PAL gene*, *unigene c14171* and *unigene c26476* refers to *CCoAOMT gene*; **b** *Unigene c26513* and *unigene c32456 * pertains to *C4H gene*, *unigene c43647* equals to *4CL gene* and *unigene c18009* was the same as *CAD gene*; **c** *Unigene c44389* and *unigene c29397* refers to *metacaspase-1-like gene*, *unigene c26668* refers to *ascorbate peroxidase gene*, *unigene c38246* belongs to *heat shock protein gene* and *unigene c26493* refers to *pathogenesis-related protein gene*, T2 and T3 stands for different stage, respectively

The KEGG pathway analysis results indicated that the phenylalanine metabolism and phenylpropaniod biosynthesis pathway also involved in this process (Fig. 5). Moreover, cinnamate-4-hydroxylase (C4H), 4-coumarate-CoA-ligase (4CL), cinnamy alcohol-dehydrogenase (CAD), caffeoyl-CoA O-methyltransferase (CCoAOMT) and phenylalanine ammonia-lyase (PAL) were the key enzymes of this pathway. The qRT-PCR results demonstrated that the *PAL* and *CCoAOMT genes* were up regulated at T2 and T3 stage, however, the *C4H*, *4CL* and *CAD genes* were down regulated (Fig. 10a, b). It means that these unigenes including *PAL* and *CCoAOMT,* which were availed for phenolic acid synthesis, were up-regulated, while the unigenes *C4H*, *C4L* and *CAD* which were related to the degradation of phenolic acid, were down-regulated. We also detected nine kinds of phenolics by HPLC from the tissue culture medium of the infected *P. heterophylla* by the pathogenic factor. The acids determined were gallic acid, cumaric acid, 3,4-dihydroxybenzoic acid, p-dihydroxybenzoic acid, vanillic acid, syringic acid, vanillin, ferulic acid, benzoic acid (Additional file 12: Figure S9). Seven of them were accumulated to a certain extent when the plantlets were induced by the pathogenic factor of *F. oxysporum*, and the concentration of vanillic acid was relatively higher than others, it also accumulated a lot after the introduction of the pathogenic factor.

### The influence of phenolic acids to the population quantity of *F. oxysporum* in the continuous cropping system of *P. heterophylla*

The mixed phenolic acids were added to the continuous cropping system of *P. heterophylla*, the quantitative analysis of the population quantity of *F. oxysporum* shows that the exogenous additives can increase the fungi amount significantly (Additional file 13: Figure S10). The amount of *F. oxysporum* in the newly planted soil added with mixed phenolic acids (NP + MPA) was much higher than the newly plant soil(NP) (Additional file 13: Figure S10). Furthermore, the population quantity of *F. oxysporum* in the two-year monoculture soil added with mixed phenolic acids (SM + MPA) was obviously bigger than the two-year monoculture soil (SM) (Additional file 13: Figure S10).

### Other important DEGs related to immunity and defense, responding against the infection of *F. oxysporum*

**Fig. 11:**
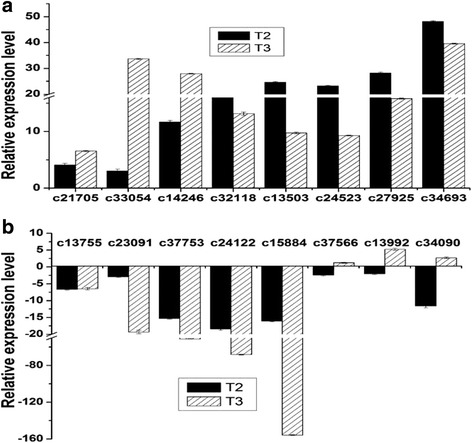
The differential expression analysis of WRKYs family unigenes by qRT-PCR. **a** The first type of unigenes include *unigene c21705* (probable WRKY transcription factor 51 like), *c33054* (WRKY transcription factor 1), *c14246* (probable WRKY transcription factor), *c32118* (WRKY transcription factor 1), *c13503* (probable WRKY transcription factor 4 like), *c24523* (probable WRKY transcription factor 20 like), *c29725* (WRKY transcription factor 1), *c34693* (probable WRKY transcription factor 32); **b** The second type of unigenes contains *unigene c28042* (WRKY transcription factor), *c15245* (WRKY transcription factor 22 like), *c13755* (probable WRKY transcription factor 20 like), *c23091* (WRKY transcription factor putative isoform 1), *c37753* (WRKY transcription factor 69), *c24122* (WRKY transcription factor 6) and *c15884* (probable WRKY transcription factor 12 like); the third type of unigenes consisted of *c37566* (probable WRKY transcription factor 74 like), *c13992* (WRKY-type transcription factor) and *c34090* (WRKY family transcription factor), T2 and T3 stands for different stage, respectively

The preliminary study on the unigenes related to programmed cell death (PCD) showed that these unigenes including *metacaspase-1-like* (*unigene c44389* and *unigene c29397*), *ascorbate peroxidase* (*unigene c26668*), *small heat shock protein (unigene c38146*) and *pathogenesis-related protein* (*unigene c26493*) *unigene* were also up-regulated both at T2 and T3 stage (Fig. 10c). The qRT-PCR results also suggested that the *WRKY transcription factor family genes* were divided into three different types: (a) the first type of unigenes were up-regulated both at T2 and T3 stage (Fig. 11a), (b) while the second type of the unigenes were completely opposite to the first type, they were down-regulated at T2 and T3 stage (Fig. 11b), (c) the third type of unigenes were down-regulated in T2 stage, but up-regulated at T3 stage (Fig. 11b), the primers of these unigenes were shown in the Additional file 10: Table S3. More information in details, the first type of unigenes includes *unigene c21705*, *c33054*, *c14246*, *c32118*, *c13503*, *c24523*, *c29725*, *c34693*. The second type of WRKY transcription factor contains *unigene c28042*, *c15245*,*c13755*, *c23091*, *c37753*, *c24122*, *c15884*. Meanwhile, *unigene c37566*, *c13992* and *c34090* belong to the third type.

## Discussion

According to the transcriptome of *P. heterophylla* responding to the infection caused by the *F. oxysporum*, the total reads from three distinct samples were assembled into 127,725 transcripts and 47,655 unigenes, and the N50 lengths of them were 2274 and 1490 (Additional file 1: Table S1), respectively. It meant that the annoated unigenes represented 54.3% of the total number of the unigenes. In similar studies of other species, such as Bamboo and *Gardenia jasminoides* Ellis, 55% and 68.6% of the total number of unigenes were annotated, respectively [15, 28]. When we take species differences and the absence of a reference genome into consideration, the assembled fragments were comparatively integrated.

Based on the COG analysis of DEGs at different stages, we found that signal transduction mechanism and transcription etc., taken over the largest group of DEGs at the early stage of the infection, which implies the signal transduction process may play an important role during the infection in the early stage. Bose et al. [29] suggested that pathogen-induced calcium ion influx occurred only in the first stages of pathogen-host plant interaction. Additionally, the KEGG analysis of DEGs also indicated that plant-pathogen interaction pathway with the highest enrichment factor may play a critical role in response to the infection (Fig. 5). Most importantly, further analysis of plant-pathogen interaction pathway showed that the DEGs in the calcium signal system were differentially expressed in this process. In our study, we found that most of the unigenes of the calcium decoding system, such as CDPKs, *CIPK*, *CML*, *CaM* were up-regulated, but some of the unigenes of the calcium encoding system (such as *Annexin* and *Glutamate receptor 3.3*) were down-regulated both at T2 and T3 stage (Figs. 6, 7), while others were not. This may suggest that the disturbing expression of the calcium signal events plays an important role in the interaction between the plants and the pathogen. Meanwhile, CDPKs are committed to perceive intracellular changes of calcium ion concentrations and translate them into specific phosphorylation events to initiate further downstream signaling processes, they played a significant role in rapid abiotic stress and immune signaling responses [30]. The changes of concentration of the calcium ion in the root tip of *P. heterophylla* suggests a calcium signal event may take part in this process. Besides, different concentrations of calcium channel blocker were used to treat with the infected plants and both of blockers can obviously weaken the expression of CDPKs and partly delay the infection process, which further confirms the function of the calcium signal system in this process.

Since the WRKY transcription factors also play an important role in the plant-pathogen interaction pathway, we further analyzed the expression pattern of all WRKY transcription factors based on our transcriptome results by using qRT-PCR. The results indicated that WRKY transcription factor family genes were divided into three different kinds of expression patterns. The first type of them were up-regulated both at T2 and T3 stage, such as *WRKY transcription factor 1/4/20/32/51*, while the second type was just reversed, *WRKY transcription factor 6/12/22/69* were down-regulated in these two stages, then the rest of them were up-regulated at T2 stage, but the reverse was true at T3 stage. A recent study has shown that WRKY transcription factors in the plant-pathogen interaction pathway are closely related to calcium signal events, and they also can be sometimes regarded as the substrates of CDPKs [31]. Meanwhile, calmodulin is a ubiquitous Ca^2+^-binding protein known to regulate diverse cellular functions by modulating the activity of various target proteins. Furthermore, one cDNA (calmodulin) encoding WRKY7 has been isolated from *Arabidopsis* as a calmodulin-binding transcription factor and it can specifically bind to the Ca^2+^-dependent calmodulin-binding domain of WRKY7 [32], suggesting that WRKYs have a close relationship with the upstream calcium signal events.

The GO analysis results suggested the amino acid transport and metabolism process obviously become more important at late stage of the infection. The KEGG results also indicated that the phenylpropaniod biosynthesis pathway plays a pivotal role in the later stage of the infection. The *PAL*, *CCoAOMT*, *C4H*, *4CL* and *CAD* genes were known as the key enzymes to participate in this pathway. The *PAL* and *CCoAOMT* gene, which were availed to synthesize some of phenolic acids were up-regulated. While the *C4H*, *C4L* and *CAD* gene, which could degrade phenolics to other kinds of compunds, were down-regulated. This result suggested that some of the phenolic acid compounds may be accumulated after the plants were infected by the pathogen. The quantitative analysis of the phenolics from the tissue culture medium of *P. heterophylla* indicated that some of pehnolic accumulated after the inducing of the pathogenic factor. Interestingly, the mixed phenolic acids promote the mycelium growth, increased as the concentration increased. And all of the phenolic acids can enhance the spore production of *F. oxysporum f.Sp. heterophylla* at a range of concentrations. Vanillin and p-hydroxybenzoic have the most obvious promoting effect under laboratory conditions [10]. Furthermore, Liu et al. [33] found that benzoic acid can promote the mycelial growth, sporulation capacity and conidial germination of the peanut root rot pathogen *Fusarium sp.* in vitro. In this study, We further confirmed that the mixed phenolic acids can promote the growth of *F. oxysporum* in the field (Additional file 13: Figure S10). That’s to say, the infection of the *F. oxysporum* may lead to the release of the phenolics which can be used by the pathogen in turn. The pathogen can make use of the phenolic acids as food for its rapid spread in the continuous cropping system of *P. heterophylla*.

Moreover, immunity is essential for plants to deal with pathogen infections. Upon infection, plant pathogenic fungi or bacteria face the plant cell wall as a first barrier. When the barrier of the plant is breached, the pathogens are recognized by plant membrane pattern recognition receptors (PRRs) through the activation of the pathogen-associated molecular pattern [34], and the second layer of immunity often culminates with a hypersensitive response (HR) programmed cell death [35]. In our research, metacaspase, ascorbate peroxidase, small heat shock protein and pathogenesis-related protein unigenes which were closely related to programmed cell death were up-regulated at T2 and T3 stage. This result suggests that programmed cell death may result in expanding obstacle of the tuber of consecutively monocultured *P. heterophylla*.

## Conclusions

The calcium signal events take part in the response process during the early stage after the infection caused by *F. oxysporum*. This process might be mediated by different WRKY transcription factors. Besides, the phenolic acids secreted by the root of *P. heterophylla* are released in rhizosphere soil, which can attract the pathogen fungal *F. oxysporum f.Sp. heterophylla* to colonize at the rooting zone. Moreover, the pathogen infection increases the synthesis pathway of phenolic acids, which can result in the accumulation of some phenolics in infected plantlets. Meanwhile, the *F. oxysporum* can make use of some of the phenolic acids as the food for its rapid spread*.* The number of the pathogen *F. oxysporum f.Sp. heterophylla* increased dramatically in the rhizosphere soil, which leads to debilitating soil health. So our findings may offer a possible explanation for the outbreak of *F. oxysporum f.Sp. heterophylla* in the rhizosphere soil of consecutive monoculture of *P. heterophylla*.

## Methods

### Total soil DNA extraction and quantitative PCR for *Fusarium oxysporum*

Our laboratory has built several observation stations near the geoauthentic growing areas of *P. heterophylla* (Zherong county, Ningde city, Fujian Province, China), all of the experimental materials were cultivated on the basis of Good Agricultural Practice (GAP) of medicinal plants standard. The newly planted *P. heterophylla* and second year planted *P. heterophylla* were grown in the same pool. The soil samples were taken from plant rhizosphere using five points sampling method. We define the soil without planting anything as the control soil (CK), the newly planted soil as NP and the two-year monoculture soil as SM. We also performed pot experiments to verify the influence of the mixed phenolic acids to the *F.oxysporum.* The ratio and concentration of mixed phenolic acids were based on the former research [12]. We respectively planted six plants in six pots in control soil and the newly planted soil in the field of our school. All of the soils were taken from the observation station. During the vigorous growth period of the *P. heterophylla* in April, we added 20 ml of mixed phenolic acids to three pots of potted plants containing the two above-mentioned different soil, the rest of them added with equal amounts of sterilized water. We started to add them from March 30th, treated them after every three days and soil samples were taken on April 29th. At this time, the plants which were planted in the control soil become the newly plant, and we define the one treated with sterilized water as newly plant soil (NP), the other one treated with mixed phenolic acids as NP + MPA. So the plants that were planted in the newly planted soil become the two-year monoculture, and we define the one treated with sterilized water as two-year monoculture soil (SM), the other one treated with mixed phenolic acids as SM + MPA. The total soil DNA was extracted by using the Bio Soil Genomic DNA Extraction Kit (Bioer, Hangzhou, China). The quantitative PCR was carried out to quantify the amount of *F. oxysporum* in different soil samples by using primers ITS1-F:CTTGGTCATTTAGAGGAAGTAA and AFP308R:CGAATTAACGCGAGTCCCAA. The quantitative PCR was performed in a 15 μl reaction mixture containing 7.5 μL of SYBR Green qPCR Master Mix (2×) (TransGen Biotech, Beijing, China), 0.6 μl of each primer (10 μM) and 1ul of template DNA (20 ng of total soil DNA or a serial dilution of plasmid DNA for standard curves).

### Plant materials preparation and treatment, RNA isolation and quality assessment

The experimental material, *P. heterophylla* of “ZheshenII”, was one of the main cultivar in Zherong country town, Fuding city, Fujian province, China. We obtained their aseptic seedlings using tissue culture technology. While the pathogen *Fusarium oxysporum* used to infect the tissue culture seedlings was isolated from the consecutively monocultured rhizosphere soil. We inoculated the pathogen hypha on the medium about 2–3 cm away from the roots of the tissue culture seedlings and chose three different stages to study the responding mechanism to the pathogen stress. The first stage, the plants without adding the *F. oxysporum* (T1), the second stage, the plants infected for 8 days (T2) and the third stage, the plants infected for 13 days (T3). We took three bottles of plantlets at each stage and there were also three plantlets in each bottle. For construction of the transcriptome library, the total RNA of each sample in different bottle from different stage was isolated using the RNAprep Pure Plant Kit (TANGEN®) following the manufacturer’s instructions respectively. All of the samples were treated with Rnase-free DNase I (TANGEN®), the concentration of the RNA was measured by the Nano Drop™ 2000C spectrophotometer (Thermo Fisher SCIENTIFIC, USA) and its integrity was analyzed by using the RNA Nano 6000 Assay Kit of the Agilent bioanalyzer 2100 system (Agilent Technologies, USA). When all of the samples satisfied the quality for sequencing. We mixed every 10 μg RNA sample from three different bottles of plantlets but on the same stage together. After that, we measured the concentration and analyzed the quality of the three samples again.

### Library preparation for Transcriptome sequencing, clustering and sequencing

Sequencing libraries were generated by using NEBNext®Ultra™ RNA Library Prep Kit for Illumina® (NEB, USA) following manufacturer’s recommendations and index codes were added to attribute sequences to each sample. A total amount of 3 μg RNA per sample was used as input material for the RNA sample preparations. Briefly, mRNA was purified from total RNA using poly-T oligo-attached magnetic beads. Fragmentation was carried out using divalent cations under elevated temperature in NEBNext First Strand Synthesis Reaction Buffer. The first strand cDNA was synthesized using random hexamer primer and M-MuLV Reverse Transcriptase. The second strand cDNA synthesis was subsequently performed using DNA Polymerase I, buffer, dNTPs and RNase H. Remaining overhangs were converted into blunt ends via exonuclease/polymerase activities. After adenylation of 3′ ends of DNA fragments, NEBNext Adaptor with hairpin loop structure were ligated to prepare for hybridization. In order to select cDNA fragments of preferentially 150–200 bp in length, the library fragments were purified with AMPure XP system (Beckman Coulter, Beverly, USA). Then 3ul USER Enzyme (NEB, USA) was used with size-selected, adaptor-ligated cDNA at 37 °C for 15 min followed by 5 min at 95 °C before the PCR. Then PCR was performed by Phusion High-Fidelity DNA polymerase, Universal PCR primers and Index (X) Primer. At last, PCR products were purified and library quality was assessed on the Agilent Bioanalyzer 2100 system. The clustering of the index-coded samples was performed on a cBot Cluster Generation System using the TruSeq PE Cluster Kit v3-cBot-HS (Illumia) according to the manufacturer’s instructions. After cluster generation, the prepared library was sequenced on an Illumina Hiseq 2000 platform and paired-end reads were generated.

### Quality control of the transcriptome sequencing and de novo assembly

Raw data (raw reads) of fastq format were firstly processed through in-house perl scripts. In this step, clean data (clean reads) were obtained by removing reads containing adapter, reads containing ploy-N and low quality reads from raw data. At the same time, Q20, Q30, GC-content and sequence duplication level of the clean data were calculated. All the downstream analyses were based on clean data with high quality. The left files from all samples were pooled into one big left.fq file, and right files into one big right.fq file. The transcriptome assembly was accomplished based on the left.fq and right.fq using Trinity [36] with min_kmer_cov set to 2 by default and all other parameters set default. The unigenes were aligned by the following several public domain databases: NCBI Nr (NCBI non-redundant nucleotide sequences); COG (Clusters of Orthologous Groups of proteins); Swiss-Prot (A manually annotated and reviewed protein sequence database); KO (KEGG Ortholog database); GO (Gene Ontology), applying a threshold E-value of 10^−5^ [37]. Gene Ontology (GO) enrichment analysis of the differentially expressed genes (DEGs) was implemented by the GOseq R packages based Wallenius non-central hyper-geometric distribution [38], which can adjust for gene length bias in DEGs; While KEGG is a database resource for understanding high-level functions and utilities of the biological system of molecular-level information [39], especially large-scale molecular datasets generated by genome sequencing and other high-throughput experimental technologies. Here we used KOBAS [40] software to test the statistical enrichment of differential expression genes in KEGG pathways.

### Screening differentially expressed genes and the qRT-PCR analysis

The differential reads from each sample were compared with the unigene library using Bowtie [41], the expression was assessed through the RSEM [42] software based on the above results. Finally, we used the RPKM value to reflect the expression density of every unigene. In addition to the *P*-value, the false discovery rate (FDR) was adjusted to identify differentially transcribed genes. Here, the thresholds for *P* value was adjusted by Q value, Q value and FDR were set at 0.005 and 0.01 [43] respectively, while the |log_2_^(Fold Change)^|≧1 was set as the threshold for significant differential expression.

To confirm the expression of the differentially expressed genes in different stages, the samples for the RNA-Seq were also used here to verify the results of the transcriptome sequencing by qRT-PCR. The total RNAs were extracted with TRIzol eagent and subjected to qRT-PCR analysis. The first strand cDNA was synthesized using the TIANScript RT Kit (TianGen Biotech, Beijing, China) in accordance with the manufacturer’s instructions, 1 μg of total RNA of each sample was converted into single stranded cDNA and the products were diluted to 80ul in DEPC water before used as templates. The reaction was performed on an Eppendorf Mastercycler realplex4 instrument (Eppendorf Mastercycler realplex4, Eppendorf, Germany) based on SYBR-Green to the detect transcript abundance, using the TransStart® Top Green qPCR SuperMix (2×) (Cat. AQ131–02). The 15 μL of the reaction system contained: 7.5 μL of SYBR Green qPCR Master Mix (2×), 0.45 μL of each of the forward and reverse primers, 0.6 μL of cDNA template and 6 μL of water. The reaction conditions were performed as follows: 95 °C for 5 min, followed by 40 cycles of 95 °C for 15 Sec, 55 °C for 20 Sec and 72 °C for 20 Sec. At least three biologically independent replicates were used for each sample. Relative gene expression levels were calculated using the 2^-ΔΔC^_T_ method [44]. Relevant PCR primers were designed by using the Primer Primer 5.0 software (Premier Biosoft, Canada) and the differentially expressed gene sequences came from the RNA-Seq results. A fragment of the gene encoding actin (*Unigene c30922*) was used as internal reference genes. we selected the stably expressed *unigene c30922* which also has a higher expression level as the reference gene, the forward primer sequence was Actin-F:5′ CTGTATTTACGCTCAGGTGG 3′ and the reverse primer sequence was Actin-R:5′ CATTGTGCTCAGTGGTGG 3′.

### The influence of calcium channel blocker to the infected *P. heterophylla*

For further study of the calcium signal system in this process, calcium channel blocker Heparin Sodium (HS, Sinopharm Chemical Reagent, Beijing, China) and Trifluoroperazine Dihydrochloride (TFP, Sigma, USA) were used to treat the plants which were infected by *F. oxysporum*. We chose six bottles of tissue culture plantlets with the fairly similar growth potential and roots flourishing (there were three plantlets in each bottle). The one without adding the *F. oxysporum* was chosen as the control, while all of the others were inoculated with the pathogen hypha on the medium as we done before. One week later, we sprayed different concentrations of calcium channel blocker solutions (1%, 2% HS and 10 mM, 20 mM TFP, respectively) to the plantlets under the infection. The one infected by the pathogen, but without spraying the blocker was named as the F.OX. The control and the F.OX were sprayed with sterile water. We sprayed all the samples twice, after 30 min of the first sprayed treatment, we sprayed the second time and took the plant samples half an hour later, then these samples were frozen in the liquid nitrogen quickly for the qRT-PCR analysis.

### The changes of calcium concentration of the *P. heterophylla* root tip after the infection of *F.oxysporum*

Another six bottles of tissue culture plantlets were chosen for this experiment, we used the liquid PDA medium to culture *F.oxysporum* at 28 °C with 180 rpm for three days. After that, we added 3 ml of liquid PDA medium to the root of the tissue culture plantlet and also added the same amount of the fungal suspension to the rest of five bottles. Two hours later, we sprayed four different kinds of calcium channel blocker to root of four bottles of tissue culture plantlets infected by *F.oxysporum*. The calcium channel blockers used were 20 mM Verapamil (Sigma, USA), 20 mM Lanthanum (III) chloride-anhydrous (LaCl_3_, Sigma, USA), 20 mM Trifluoroperazine Dihydrochloride (TFP, Sigma, USA) and 2% Heparin Sodium (HS, Sinopharm Chemical Reagent, Beijing, China). Therefore, the four samples were named after the four different kinds of treatments. Meanwhile, we also sprayed the rest of two bottle with the same amount of sterile water. The one without adding the fungal suspension, but sprayed with sterile water was chosen as the control (CK), the other one infected by *F.oxysporum* but without spraying the blocker was named as the F.OX. We sprayed all the plantlets twice, after 30 min of the first spray treatment, we sprayed the second time and took the root tip sample 30 min later.

The root tip samples were put into the incubation solution which included 0.2 mM CaC1_2_, 50 mM sorbitol, 20 μm Fluo-3 AM (Beyotime Biotechnology, China), and the final concentration of dimethylsulfoxide in this solution were 1% (V/V). All the samples were wrapped in foil and incubated in the constant temperature incubator at 4 °C for two hours, then the samples were taken out and washed twice with 0.2 mM CaC1_2_ solution, after that, all the samples were put into 0.2 mM CaC1_2_ solution, wrapped in foil and incubated in the constant temperature incubator at room temperature for two hours [45]. At last, all of the samples were made into temporary a slide and observed under ten times fluorescence microscope (Nikon, Ni-U, Japan).

### The preparation of the pathogenic factor from the cell wall of *F. oxysporum*

We inoculated the *Fusarium oxysporum* to PDA medium and cultured at 28 °C for three days, then we added a small pieces (about 1cm^2^) of this activated fungi strain into the 150 ml liquid PDA medium and cultured in the shaker at 28 °C with 120 rpm for 5 days. After that, we filtered and gathered the hypha, put 10 g of the them into 1 L double distilled water (PH 2.0) and boiled for 1 h. Then, we filtered and collected the filtered liquor, adjusted the PH to 5.0 and supplemented double distilled water to 1 L before we use.

### The extraction of phenolics from the tissue culture medium of *P. heterophylla* affected by the pathogenic factor and there determination of them by HPLC

Six bottles of tissue culture plantlets (three plantlets in each bottle) were chosen for this experiments. We added 5 ml of pathogenic factor into three bottles of them on the MS medium. In the same way, 5 ml sterile double distilled water were added to the rest of other three bottles. Then, the tissue cultures of *P. heterophylla* were incubated for six months at 25 °C, 2000Lux. The extraction and determination methods of phenolics were referenced as we previously described [12]. Briefly, we used 1 M NaOH solution to extract the phenolics and detected them by HPLC.

### Statistical analysis

Differences among the treatments were calculated and statistically analyzed using the analysis of variance (ANOVA) and the LSD multiple range test (*p* < 0.05). The Statistical Package for the GraphPad Prism version 5.1 and the Data Processing System (DPS) version 7.05 were used for statistic analysis.

## Additional files

Additional file 1: Table S1. Summary of the assembly statistics. (DOCX 14 kb)

Additional file 2: Table S2. The annotated unigenes statistics analysis. (DOCX 13 kb)

Additional file 3: Figure S1. Gene Ontology classification of the DEGs of different stages. The left side and the right side of the panel show the percentage of genes and the number of genes that are classified in the corresponding term, respectively. (TIFF 3485 kb)

Additional file 4: Figure S2. COG annotation of the DEGs between T1 and T2. The left side and the right side of the graph show the frequency number and the classified COG categories, respectively. (TIFF 617 kb)

Additional file 5: Figure S3. COG annotation of the DEGs between T1 and T3. The left side and the right side of the graph show the frequency number and the classified COG categories, respectively. (TIFF 617 kb)

Additional file 6: Figure S4. COG annotation of the DEGs between T2 and T3. The left side and the right side of the graph show the frequency number and the classified COG categories, respectively. (TIFF 621 kb)

Additional file 7: Figure S5. Venn diagram of DEGs in three pairs of different development stages. The differential expression genes between stage T1 and stage T2 (T1 VS T2); the differential expression genes between stage T1 and stage T3 (T1 VS T3); the differential expression genes between stage T2 and stage T3 (T2 VS T3). (TIFF 600 kb)

Additional file 8: Figure S6. Verification of the differential expression genes screening of the RNA-Seq results by Real-time quantitative PCR. The lines with triangle indicate the qRT-PCR results with the “2(−∆∆CT)” value corresponding to the primary axis on the left side and the lines with dot show the RNA-Seq results with the RPKM value corresponding to the secondary axis on the right side. The expression quantity of all the unigenes was set as one at T1 stage, and T1, T2, T3 stands for different stage respectively. (TIFF 1118 kb)

Additional file 9: Figure S7. The KEGG pathway of the DEGs in the Plant-Pathogen interaction. These genes marked with red colour indicate that they are up-regulated and the green colour means these genes are down-regulated, those genes labeled with blue colour refer to some of the unigenes are up-regulated, while others are down regulated. All of the DEGs at T2 and T3 stage contrast with these genes at T1 stage. (TIFF 421 kb)

Additional file 10: Table S3. The Real-time quantitative PCR primers of the differential expression unigenes. (DOCX 15 kb)

Additional file 11: Figure S8. Calcium signal related unigenes response to the infection of the pathogen. The *Calcium dependent protein kinase gene* includes unigene c26726, c19133, c31289, c13860; *CBL-interacting protein kinase gene* contains c33227, c31920, c33951, c13791; *Calcium binding protein gene* contains c17699,c36329,c17689, c35958; *Glutamate receptor gene* includes c35532, c32236, c28906, c35578; *Annexin gene* includes c13258, c27953, c36074, c26955; *Calcium transporting ATPase gene* contains unigene c29327, c33245, c32998, c35597. (TIFF 1596 kb)

Additional file 12: Figure S9. Quantitative analysis of the phenolics from the tissue culture medium of *P. heterophylla*. CK: the plantlets treated with sterile double distilled water, Pathogenic factor of F.OX: the plantlets affected by the pathogenic factor. (TIFF 387 kb)

Additional file 13: Figure S10. Quantification of *F. oxysporum* after the addition of the mixed phenolic acids to soil. NP: the newly planted soil, NP + MPA: the newly planted soil added with mixed phenolic acids, SM: the two-year monoculture soil, SM + MPA: the two-year monoculture soil added with mixed phenolic acids. Different superscripts in the column indicate significant differences with each other (*P* ≤ 0.01,*n* = 4). (TIFF 2103 kb)

## Abbreviations

4CL: 4-coumarate-CoA-ligase
C4H: Cinnamate-4-hydroxylase
CAD: Cinnamy alcohol-dehydrogenase
CaM: Ca^2+^ sensors like Calmodulins
CCoAOMT: Caffeoyl-CoA-O-methyltransferase
CDPK: Calcium dependent protein kinase
CML: Calcium-binding protein
COG: Clusters of Orthologous Groups
DEGs: Differentially expressed unigenes
DGGE: Denaturing gradient gel electrophoresis
FLS2: LRR receptor-like serine/threonine-protein kinase FLS2
GO: Gene Ontology
HS: Heparin sodium
Hsp: Heat shock protein
KEGG: Kyoto Encyclopedia of Genes and Genomes
PAL: Phenylalanine ammonia-lyase
PCD: Programmed cell death
qRT-PCR: quantitative real time PCR
RNA-Seq: RNA sequencing
T1: The first stage the plants without adding the *Fusarium oxysporum*
T2: The second stage with the plants infected for 8 days
T3: The third stage with the plants infected for 13 days
TFP: Trifluoperazine
