# Supplementary material for: Transcriptome analysis of Pseudostellaria heterophylla in response to the infection of pathogenic Fusarium oxysporum
Source: BMC Plant Biol. 2017 Sep 18;17:155. doi: 10.1186/s12870-017-1106-3 (PMC5604279; doi:10.1186/s12870-017-1106-3)
Supplement: Supplementary file 1 — Summary of the assembly statistics. (DOCX 14 kb) [file 12870_2017_1106_MOESM1_ESM.docx]

| Length range | Contigs | Transcripts | Unigenes |
| --- | --- | --- | --- |
| 200-300 | 2,497,004(98.35%) | 17,907(14.07%) | 14,764(30.98%) |
| 300-500 | 16,320(0.64%) | 17,121(13.40%) | 11,936(24.63%) |
| 500-1000 | 12,151(0.48%) | 21,883(17.13%) | 8,846(18.56%) |
| 1000-2000 | 9,044(0.36%) | 35,334(27.66%) | 7,637(16.03%) |
| 2000+ | 4,382(0.17%) | 35,480(27.78%) | 4,672(9.80%) |
| Total number | 2,583,941 | 127,125 | 47,655 |
| Total length | 146,864,157 | 190,404,312 | 39,504,858 |
| N50 length | 49 | 2,274 | 1,490 |
| Mean length | 57.84 | 1490.74 | 828.90 |

**Table S1** Summary of the assembly statistics.
