# Supplementary material for: Transcriptome analysis of Pseudostellaria heterophylla in response to the infection of pathogenic Fusarium oxysporum
Source: BMC Plant Biol. 2017 Sep 18;17:155. doi: 10.1186/s12870-017-1106-3 (PMC5604279; doi:10.1186/s12870-017-1106-3)
Supplement: Supplementary file 2 — The annotated unigenes statistics analysis. (DOCX 13 kb) [file 12870_2017_1106_MOESM2_ESM.docx]

| Annotated databases | All sequence | >=300bp | >=1000bp |
| --- | --- | --- | --- |
| COG | 8,240 | 7,472 | 5,099 |
| GO | 19,123 | 16,539 | 9,348 |
| KEGG | 5,836 | 5,050 | 2,982 |
| Swiss-Prot | 17,102 | 14,969 | 8,484 |
| NR | 25,791 | 22,071 | 11,654 |
| All | 25,882 | 22,125 | 11,657 |

**Table S2** The annotated unigenes statistics analysis.
