# Supplementary figures and images for: Transcriptome analysis of Pseudostellaria heterophylla in response to the infection of pathogenic Fusarium oxysporum
Source: BMC Plant Biol. 2017 Sep 18;17:155. doi: 10.1186/s12870-017-1106-3 (PMC5604279; doi:10.1186/s12870-017-1106-3)

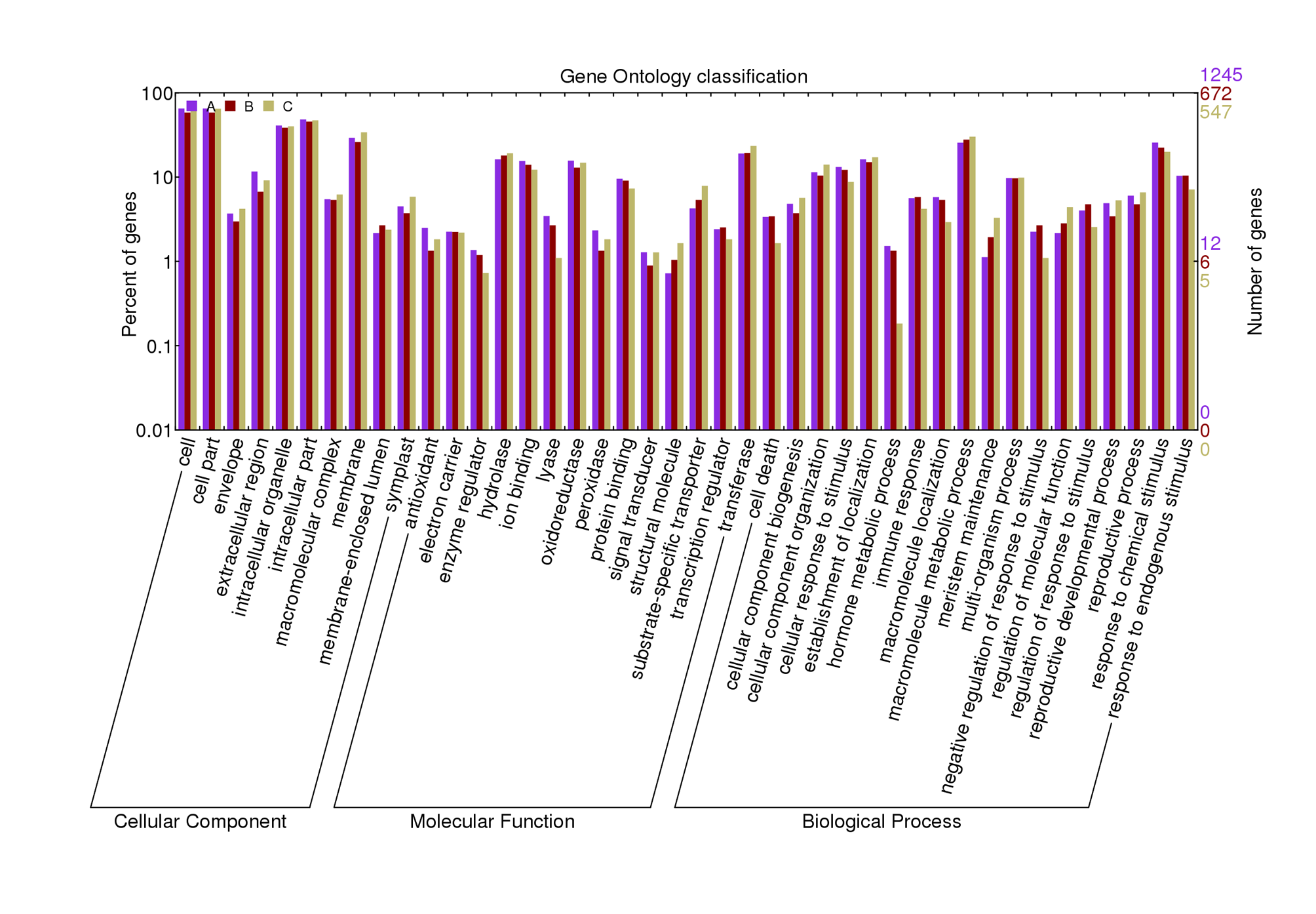

Supplement: Supplementary file 3 — Gene Ontology classification of the DEGs of different stages. The left side and the right side of the panel show the percentage of genes and the number of genes that are classified in the corresponding term, respectively. (TIFF 3485 kb) [file 12870_2017_1106_MOESM3_ESM.tif]

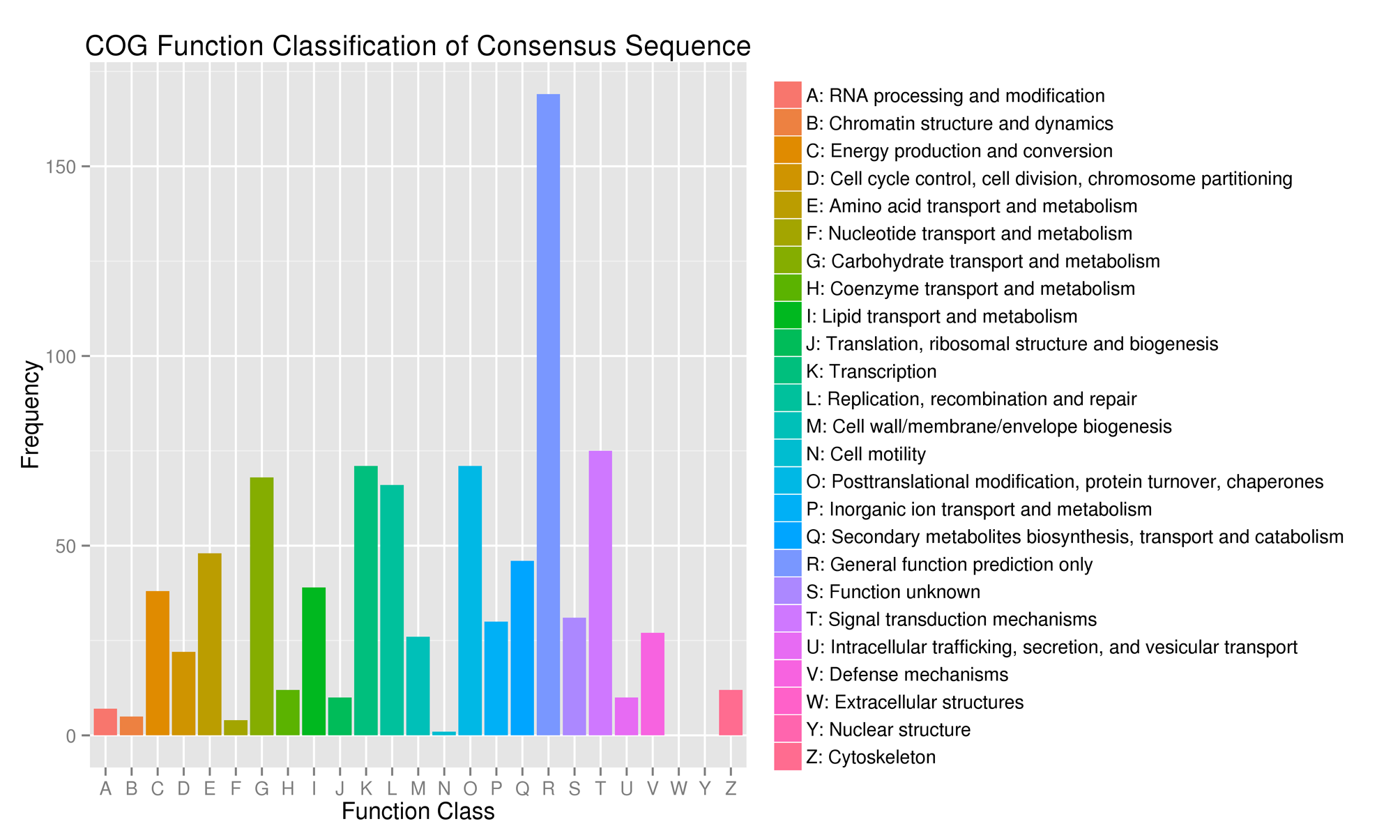

Supplement: Supplementary file 4 — COG annotation of the DEGs between T1 and T2. The left side and the right side of the graph show the frequency number and the classified COG categories, respectively. (TIFF 617 kb) [file 12870_2017_1106_MOESM4_ESM.tif]

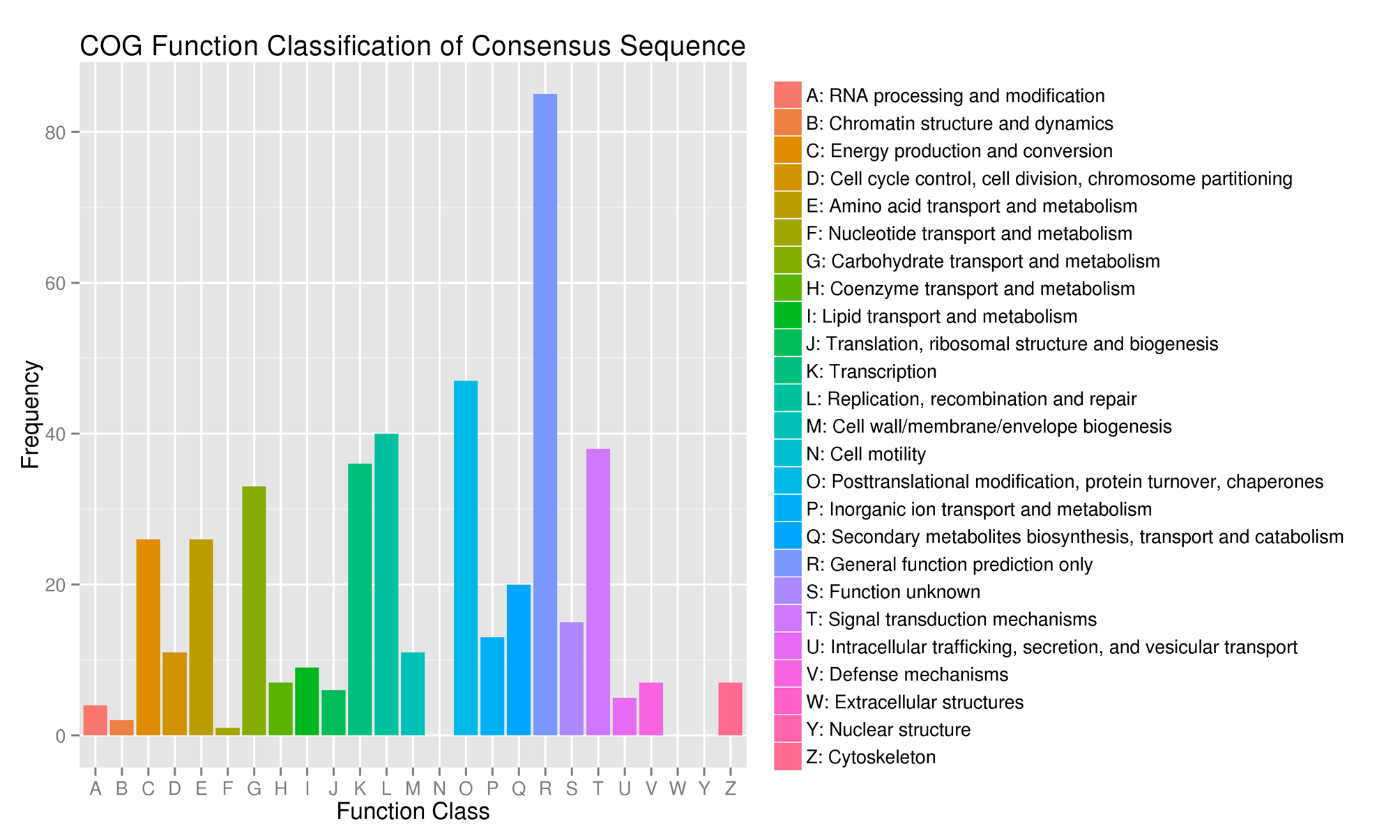

Supplement: Supplementary file 5 — COG annotation of the DEGs between T1 and T3. The left side and the right side of the graph show the frequency number and the classified COG categories, respectively. (TIFF 617 kb) [file 12870_2017_1106_MOESM5_ESM.tif]

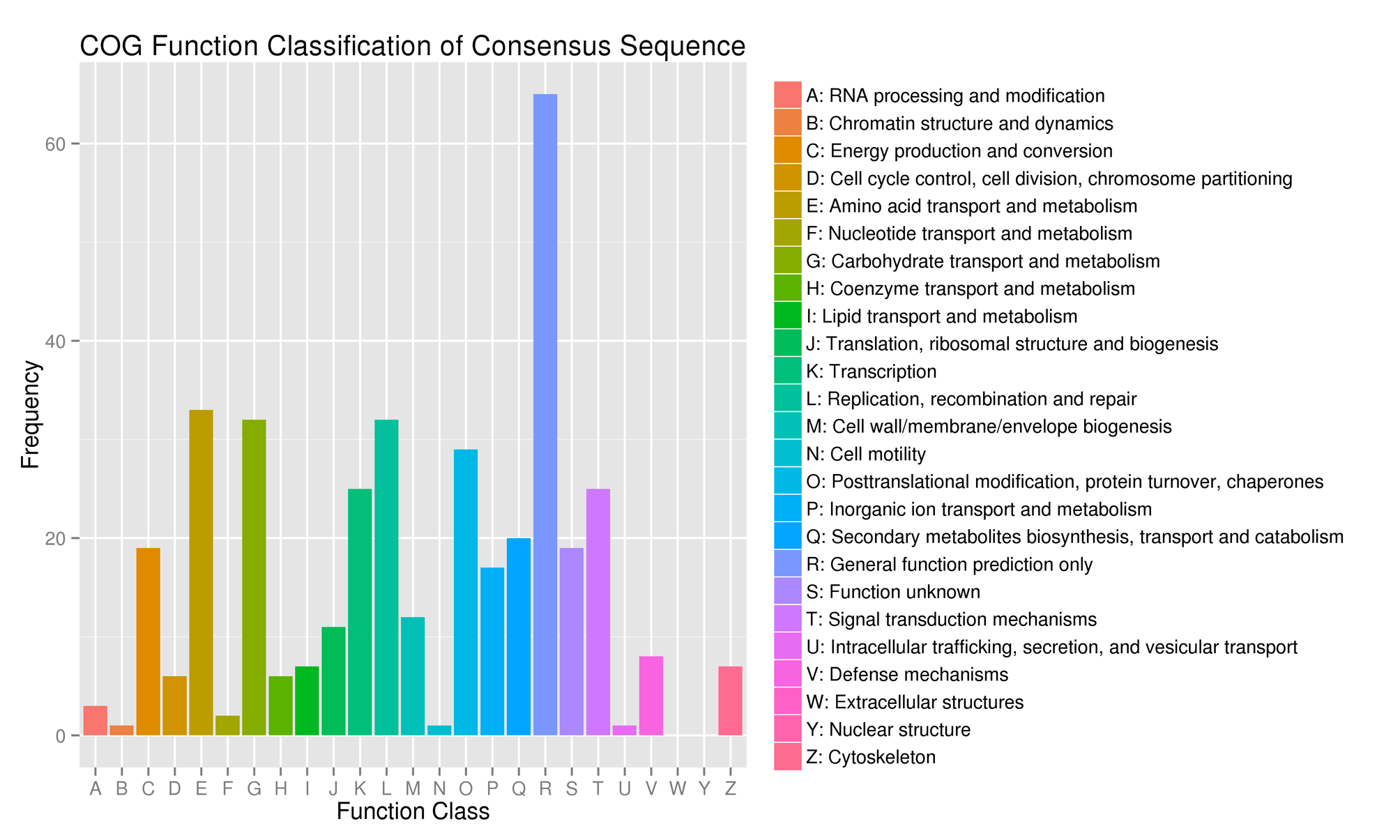

Supplement: Supplementary file 6 — COG annotation of the DEGs between T2 and T3. The left side and the right side of the graph show the frequency number and the classified COG categories, respectively. (TIFF 621 kb) [file 12870_2017_1106_MOESM6_ESM.tif]

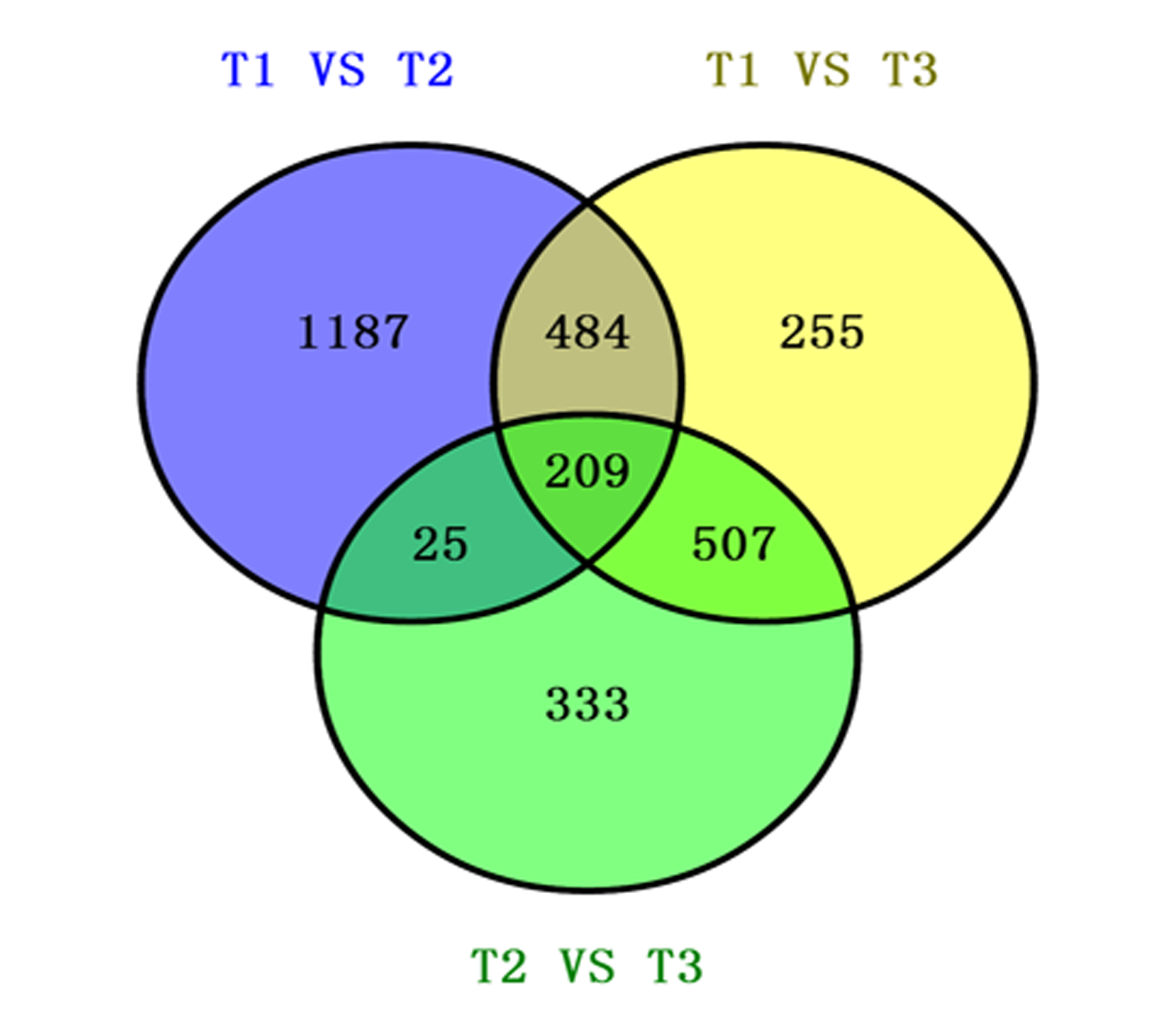

Supplement: Supplementary file 7 — Venn diagram of DEGs in three pairs of different development stages. The differential expression genes between stage T1 and stage T2 (T1 VS T2); the differential expression genes between stage T1 and stage T3 (T1 VS T3); the differential expression genes between stage T2 and stage T3 (T2 VS T3). (TIFF 600 kb) [file 12870_2017_1106_MOESM7_ESM.tif]

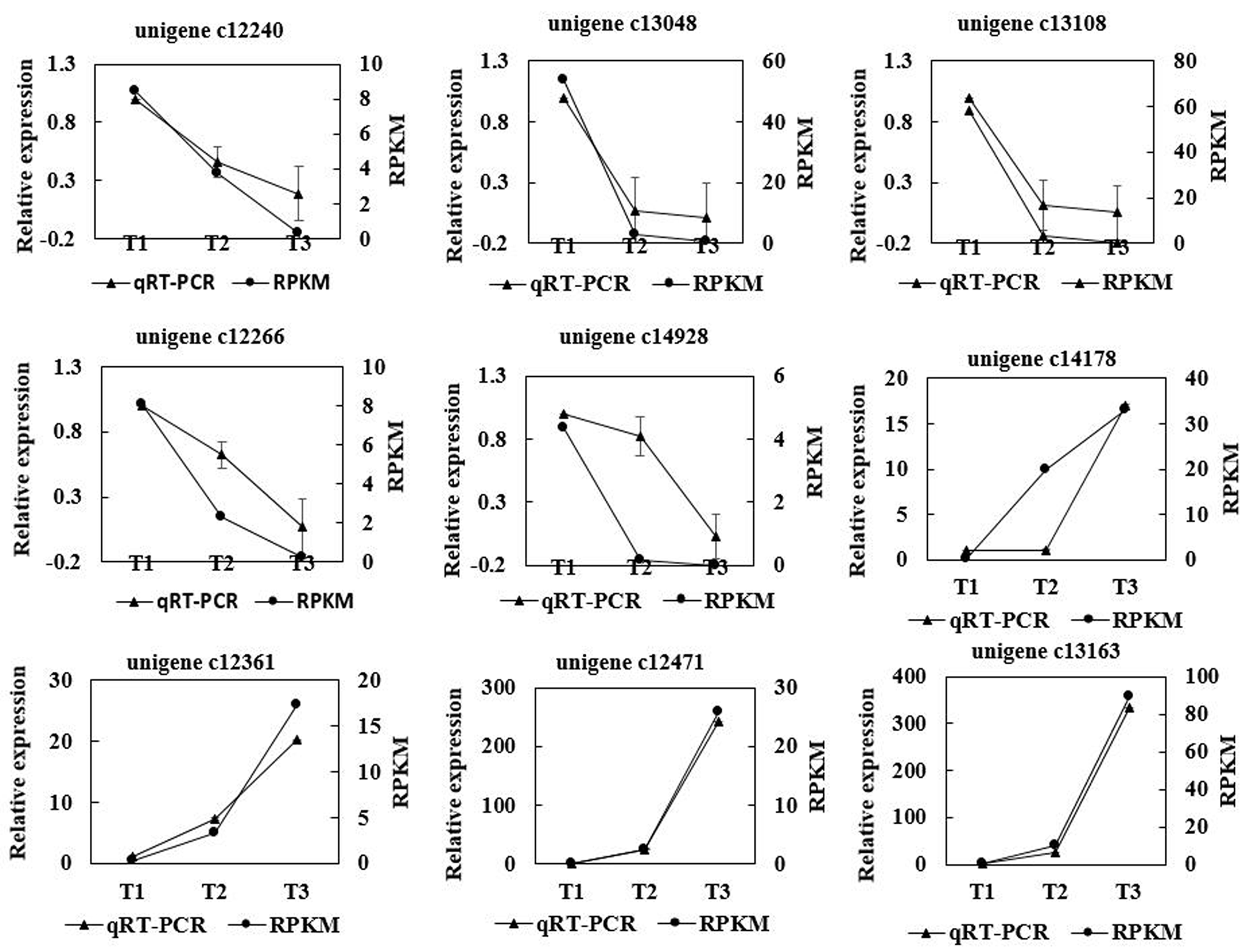

Supplement: Supplementary file 8 — Verification of the differential expression genes screening of the RNA-Seq results by Real-time quantitative PCR. The lines with triangle indicate the qRT-PCR results with the “2(−∆∆CT)” value corresponding to the primary axis on the left side and the lines with dot show the RNA-Seq results with the RPKM value corresponding to the secondary axis on the right side. The expression quantity of all the unigenes was set as one at T1 stage, and T1, T2, T3 stands for different stage respectively. (TIFF 1118 kb) [file 12870_2017_1106_MOESM8_ESM.tif]

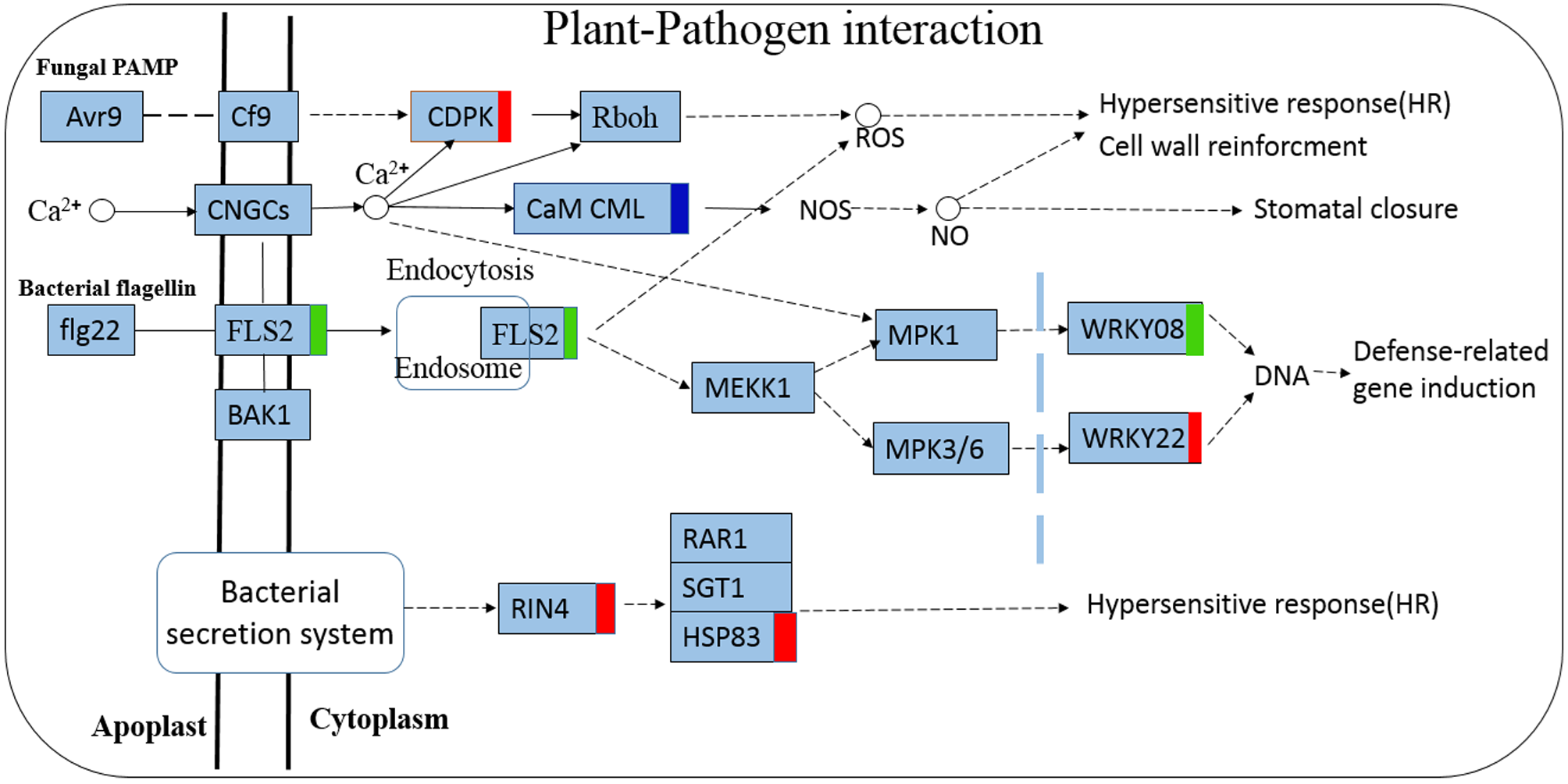

Supplement: Supplementary file 9 — The KEGG pathway of the DEGs in the Plant-Pathogen interaction. These genes marked with red colour indicate that they are up-regulated and the green colour means these genes are down-regulated, those genes labeled with blue colour refer to some of the unigenes are up-regulated, while others are down regulated. All of the DEGs at T2 and T3 stage contrast with these genes at T1 stage. (TIFF 421 kb) [file 12870_2017_1106_MOESM9_ESM.tif]

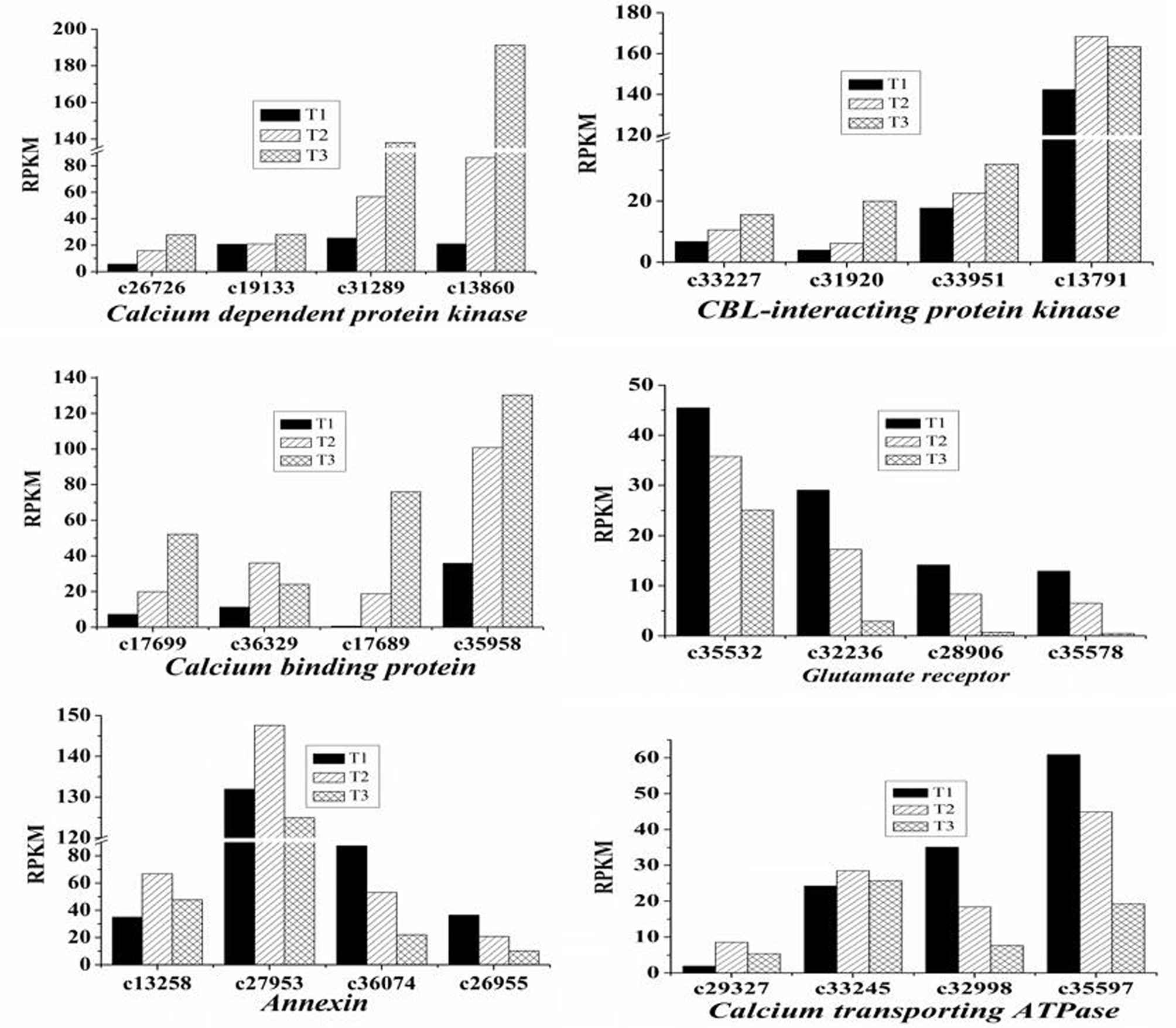

Supplement: Supplementary file 11 — Calcium signal related unigenes response to the infection of the pathogen. The Calcium dependent protein kinase gene includes unigene c26726, c19133, c31289, c13860; CBL-interacting protein kinase gene contains c33227, c31920, c33951, c13791; Calcium binding protein gene contains c17699,c36329,c17689, c35958; Glutamate receptor gene includes c35532, c32236, c28906, c35578; Annexin gene includes c13258, c27953, c36074, c26955; Calcium transporting ATPase gene contains unigene c29327, c33245, c32998, c35597. (TIFF 1596 kb) [file 12870_2017_1106_MOESM11_ESM.tif]

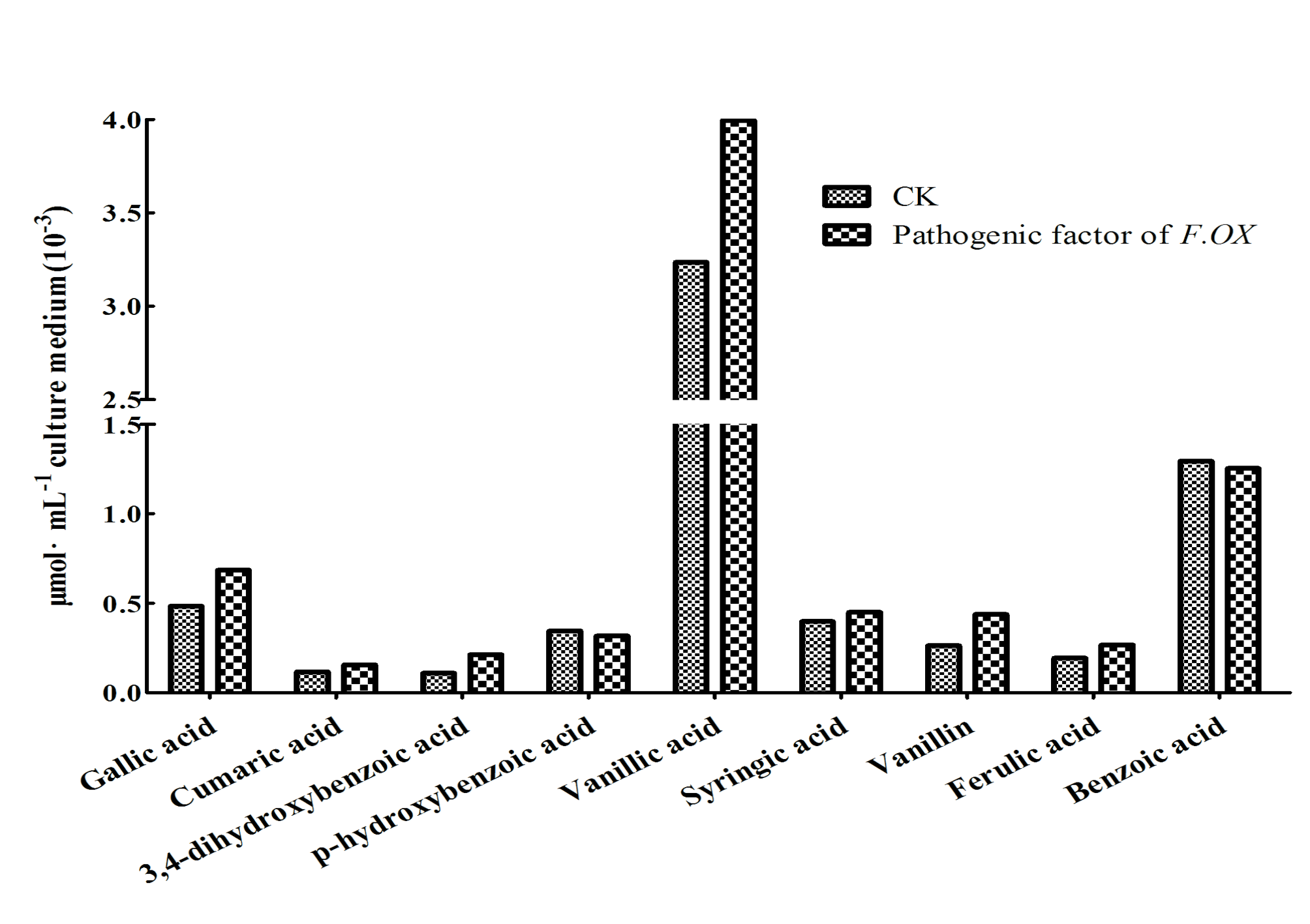

Supplement: Supplementary file 12 — Quantitative analysis of the phenolics from the tissue culture medium of P. heterophylla. CK: the plantlets treated with sterile double distilled water, Pathogenic factor of F.OX: the plantlets affected by the pathogenic factor. (TIFF 387 kb) [file 12870_2017_1106_MOESM12_ESM.tif]
