# Supplementary material for: Transcriptome analysis of Pseudostellaria heterophylla in response to the infection of pathogenic Fusarium oxysporum
Source: BMC Plant Biol. 2017 Sep 18;17:155. doi: 10.1186/s12870-017-1106-3 (PMC5604279; doi:10.1186/s12870-017-1106-3)
Supplement: Supplementary file 10 — The Real-time quantitative PCR primers of the differential expression unigenes. (DOCX 15 kb) [file 12870_2017_1106_MOESM10_ESM.docx]

**Table S3** The Real-time quantitative PCR primers of the differential expression unigenes.

| Forward primer | Reverse primer |
| --- | --- |
| Unigene c12240-F:5' TTTCCCAAAGCCAGACCT 3' | Unigene c12240-R:5' CATCAAACCTATGCCGACC 3' |
| Unigene c13108-F:5' ACCTGAGAACAGCGAAGATGC 3' | Unigene c13108-R:5' GGTGAATACTCCCTCGGAATAGA 3' |
| Unigene c13048-F:5' GAAGGGGTAACCTATGTCC 3' | Unigene c13048-R:5' TCCGCCTGTAAAGAAGAG 3' |
| Unigene c12266-F:5' TCCGAACCCCGAACTAAC 3' | Unigene c12266-R:5' CGACCCTATGACGAACACTC 3' |
| Unigene c14928-F:5' GTGGGGTGGAATGGACTTTG 3' | Unigene c14928-R:5' GGCACTGAACAAGCCAACGA 3' |
| Unigene c14178-F:5' CCTTGGTCTCCTCGTTGTCTG 3' | Unigene c14178-R:5' TTGTAATCGGCTGGCTTGG 3' |
| Unigene c12361-F:5' ATGAACAATCAAGAAGGTTTATGGT 3' | Unigene c12361-R:5' TTCTGACGTACGAGCTTCCACTAGG 3' |
| Unigene c12471-F:5' ACGGAGATTCGGGACCAT 3' | Unigene c12471-R:5' GTCAATCTTGCCGTTAGGGT 3' |
| Unigene c13163-F:5' CCAAGCCAACCTCGATAAAC 3' | Unigene c13163-R:5' TGATTTCAGCGGCGGTCTC 3' |
| Unigene c21551-F:5' ATGAACCCGTCTCCATCAGTATC 3' | Unigene c21551-R:5' ACGAATGGCGATGGCAAG 3' |
| Unigene c13860-F: 5'TTGAGCAAAGGTATGAACTGGG 3' | Unigene c13860-R: 5'GGGTGACGATGTTAGGGTGG 3' |
| Unigene c17689-F:5' TGTAAGAGCGGTAGGAGGGC 3' | Unigene c17689-R:5' TCGTCGCAGTTTCACCAAGT 3' |
| Unigene c35958-F:5' GAAGCAGAGGCAGCAGTAGAGT 3' | unigene c35958-R:5' GCCGTAATAACCCCTGAACC 3' |
| Unigene c25381-F:5' GACTGGCATATCCTGTGACCC 3' | Unigene c25381-R:5' CGAGCCCGAAAGATTGTTGT 3' |
| Unigene c32450-F:5' TTACCACCAAGCACAATGACG 3' | Unigene c32450-R:5' TCTCAGTCCAGAGGTAGATAGGGT 3' |
| Unigene c13196-F:5' GTTTGGTAGTTGGAATGGCG 3' | Unigene c13196-R:5' TTCTCCTGACCTCTTTGGCTC 3' |
| Unigene c34546-F:5' GGCGGACATTGGTGTTTCA 3' | Unigene c34546-R:5' GCTTCATGGCTGCGAGTTAG 3' |
| Unigene c13992-F:5' ATCTTCTGATATGTGGGCATGG 3' | Unigene c13992-R:5' AAGCGAGTTACGGTGAGTTGG 3 |
| Unigene c26955-F:5' GCAAGTAACGAGCCTGAT 3' | Unigene c26955-R:5' TCGATGGATGTTGGACC 3' |
| Unigene c32527-F:5'GCACCCGATATGTCCACT 3' | Unigene c32527-R:5' GCGTCCTAATCCGTTTCA 3' |
| Unigene c14000-F:5' AAAGACCTCCCTACCATCG 3' | Unigene c14000-R:5' CCCTTCACAAAGCTCCAT 3' |
| Unigene c31289-F:5' GGGGCATTACTCTGAACG 3' | Unigene c31289-R:5' CCAACAAGGTCTCGGAAG 3' |
| Unigene c19133-F:5' GAGGTGGATATTTGGAGTGC 3' | Unigene c19133-R:5' CGCTTCTTAGGGTCTGGTT 3' |
| Unigene c343755-F :5' GGGTGCTTATGAGGATGC 3' | Unigene c34375-R: 5' CGATAGCTTTGAGGGAGA 3' |
| Unigene c26726-F:5' ATGGAGCCCCTTTCTACC 3' | Unigene c26726-R:5' CCATCGGAACTTCGTATC 3' |
| CIPK-F:5' TCGGCTTTAGCACCATCAT 3' | CIPK-R:5' TGGGAATGTCAAGGTCTCG 3' |
| CaM-F:5' GGHTGYATYACHACHAAGGARCT 3' | CaM-R:5' ATYTGDCCRTCRCCRTCVACRTC 3' |
| Unigene c28761-F:5' AATAGTGCCGTGCTTTCG 3' | Unigene c28761-R:5' AGCCAACCCTGCTTTTAG 3' |
| Unigene c17725-F:5'  GAACAACAGCAACCACTCATCA  3' | Unigene c17725-R:5'  CTCTGTCCTTAGCAGCCTCATT  3' |
| Unigene c32464-F:5' TATCACCACCTCCATCTTCC 3' | Unigene c32464-R:5' TCCTTCACCTGTCAGCAACT 3' |
| Unigene c14171-F:5' AGGACCTACTAAATCAGGGCAAAG 3' | Unigene c14171-R:5' CGAATTGTTCATCGTCGTCTG 3' |
| Unigene c26476-F:5' GCTACTGCCCTTGCTCTTCC 3' | Unigene c26476-R:5' CGTACCCGATAACACCACCA 3' |
| Unigene c26513-F:5' AATGGGGAATAGCGGAGTTAG 3' | Unigene c26513-R:5' AGTGGGCTGGATTGTTTGC 3' |
| Unigene c32456-F:5' TTTTGCCATTTGGTGTCGG 3' | Unigene c32456-R:5' CATGATGAACGGGTTTGAAGAC 3' |
| Unigene c43647-F:5' GCCACTGGTATCTCCCCTGCTT 3' | Unigene c43647-R:5' TCCTGCTGTCTCACCCTTCG 3' |
| Unigene c18009-F:5' GGCCGCGAAATCCATCATCT 3' | Unigene c18009-R:5' GGAGTTACCAATCTTCCCTCTGCT 3' |
| Unigene c38146-F:5' GAAGAAGGTGGAGACCAAGAACG 3' | Unigene c38146-R:5' CCATCCAACCACGAGATAGCC 3' |
| Unigene c26668-F:5' TGCCACAGTGCCTTTCTTTAC 3' | Unigene c26668-R:5' CCAGCAGCCTTCCCTTTAGTC 3' |
| Unigene c29397-F:5' GAATGAGCAGGAGTGGACAGTATG 3' | Unigene c29397-R:5' CGTCGCAGCCGCTAAAAGA 3' |
| Unigene c44389-F:5' GCTTGAAGGATTTGACGAGAC 3' | Unigene c44389-R:5' TCGATGATTGCGTGAAGTGTC 3 |
| Unigene c26493-F:5' TCCTGCCTCGGTTGGATGT 3' | Unigene c26493-R:5' CTCCCTTGGTATGGTACTCTGC 3' |
| Unigene c21705-F:5'  CCAAGTGAGCAAGAGTCTGTG  3' | Unigene c21705-R:5'  GACCTCATTCTGAACGCAAC  3' |
| Unigene c33054-F:5'  CCAGTGGAGAAAATACGGTCA  3' | Unigene c33054-R:5'  CAAGGTTGGAGTGGATGTCA  3' |
| Unigene c14246-F:5'  AACAGCCTCAAAAGGGTCA  3' | Unigene c14246-R:5'  CGTTCGAGTTTTCGGGAC  3' |
| Unigene c32118-F:5'  TGGAGGAAATACGGTCAAAAGG  3' | Unigene c32118-R:5'  CCGAACCAACGGTGAAAAC  3' |
| Unigene c13503-F:5'  AGCAGGAATCACAAATAGCG  3' | Unigene c13503-R:5'  GGGTTCTGATACTGTCCGATG  3' |
| Unigene c24523-F:5'  GACGCATCCCACTTACATCG  3' | Unigene c24523-R:5'  GGTTCTCGGATAGGCTTGAC  3' |
| Unigene c27925-F:5'  ATCCGAATCCCAGGAGTTAC  3' | Unigene c27925-R:5'  TAATGGTGCGGGGTCAGT  3' |
| Unigene c34693-F:5'  GAAGGTGTTGTTTTGGCTGCTG  3' | Unigene c34693-R:5'  CGGGTGACTTGGGTGGATTA  3' |
| Unigene c13755-F:5'  ATGGAAGCACCACCGTCA  3' | Unigene c13755-R:5'  AGCCAGTAGTTGGGGAAGG  3' |
| Unigene c23091-F:5'  TGCACCATTGTCCACCATAC  3' | Unigene c23091-R:5'  GTCCAACTCGGCATCTTCTT  3' |
| Unigene c37753-F:5'  GGATGATTCCTTGTTTGCTG  3' | Unigene c37753-R:5'  CGTGTCTGGTGGTGTTGCTC  3' |
| Unigene c24122-F:5'  CCCCTATGATAAGCGATGG  3' | Unigene c24122-R:5'  ACGGACAGGACAAGCAGTG  3' |
| Unigene c37566-F:5'  GGATGCTAGTGTTGCTGGTG  3' | Unigene c37566-R:5'  CCCCTTAATCGGCTTCTG  3' |
| Unigene c13992-F:5'  TGCAGCCTTCCACCAATC  3' | Unigene c13992-R:5'  CATCAAACTCATCCCCGAC  3' |
| Unigene c34090-F:5'  AGCAAAGCCAGTAGCATC  3' | Unigene c34090-R:5'  TATTGGGCTATGTGAGGC  3' |
